# The Glutamate–Glutamine (GABA) Cycle: Importance of Late Postnatal Development and Potential Reciprocal Interactions between Biosynthesis and Degradation

**DOI:** 10.3389/fendo.2013.00059

**Published:** 2013-05-27

**Authors:** Leif Hertz

**Affiliations:** ^1^Clinical Pharmacology, Medical University of ChinaShenyang, China

**Keywords:** aspartate aminotransferase, glutamine–glutamate cycle, postnatal metabolic enzyme development

## Abstract

The gold standard for studies of glutamate–glutamine (GABA) cycling and its connections to brain biosynthesis from glucose of glutamate and GABA and their subsequent metabolism are the elegant *in vivo* studies by ^13^C magnetic resonance spectroscopy (NMR), showing the large fluxes in the cycle. However, simpler experiments in intact brain tissue (e.g., immunohistochemistry), brain slices, cultured brain cells, and mitochondria have also made important contributions to the understanding of details, mechanisms, and functional consequences of glutamate/GABA biosynthesis and degradation. The purpose of this review is to attempt to integrate evidence from different sources regarding (i) the enzyme(s) responsible for the initial conversion of α-ketoglutarate to glutamate; (ii) the possibility that especially glutamate oxidation is essentially confined to astrocytes; and (iii) the ontogenetically very late onset and maturation of glutamine–glutamate (GABA) cycle function. Pathway models based on the functional importance of aspartate for glutamate synthesis suggest the possibility of interacting pathways for biosynthesis and degradation of glutamate and GABA and the use of transamination as the default mechanism for initiation of glutamate oxidation. The late development and maturation are related to the late cortical gliogenesis and convert brain cortical function from being purely neuronal to becoming neuronal-astrocytic. This conversion is associated with huge increases in energy demand and production, and the character of potentially incurred gains of function are discussed. These may include alterations in learning mechanisms, in mice indicated by lack of pairing of odor learning with aversive stimuli in newborn animals but the development of such an association 10–12 days later. The possibility is suggested that analogous maturational changes may contribute to differences in the way learning is accomplished in the newborn human brain and during later development.

## Glutamate and GABA

The function of glutamate and γ-aminobutyric acid (GABA) as the key excitatory and inhibitory transmitters in mammalian brain was not realized until the second half of the twentieth century (Okamoto, 1951; Florey, 1956; Roberts, 1956; Curtis et al., 1960; Watkins, 2000). Relatively soon thereafter evidence was obtained that a cycle of neuronal-astrocytic interactions plays a major role in the production from glucose and the metabolism of both amino acid transmitters (van den Berg and Garfinkel, 1971; Benjamin and Quastel, 1972), and intense uptake of the transmitters, especially glutamate, was demonstrated and quantitated in astrocytic preparations (McLennan, 1976; Schousboe et al., 1977; Hertz et al., 1978a,b). Although the importance of glutamatergic/GABAergic activation of endocrine responses was suggested already at that time (Ondo and Pass, 1976; Ondo et al., 1976), the full consequence of the involvement of the amino acid transmitters only became realized during the last decade. More recently, direct evidence is emerging that astrocytes may also account for much of glutamate degradation (Bauer et al., 2012; McKenna, 2012; McKenna, present Research Topic; Whitelaw and Robinson, present Research Topic), and that production and degradation pathways may interact (Hertz, 2011a). These recent conclusions and observations place an increased focus on identification of the enzymes(s) carrying out the undisputed initial conversion of glutamate to α-ketoglutarate (α-KG) (almost certainly mainly transamination) and, especially *vice-versa*. This paper will deal with these questions and discuss a possible interaction between the pathways mediating synthesis and degradation of the two amino acid transmitters. It will also discuss an observed late maturation of the metabolic processes involved. Many of the developmental observations were made decades ago, but their full importance can only now be understood after the realization in the living rodent and human brain of the huge glutamine–glutamate (GABA) cycle flux determined in the brain *in vivo* and described below.

## The Glutamine–Glutamate (GABA) Shuttle and Its Relation to Glucose Metabolism

**Figure 1:**
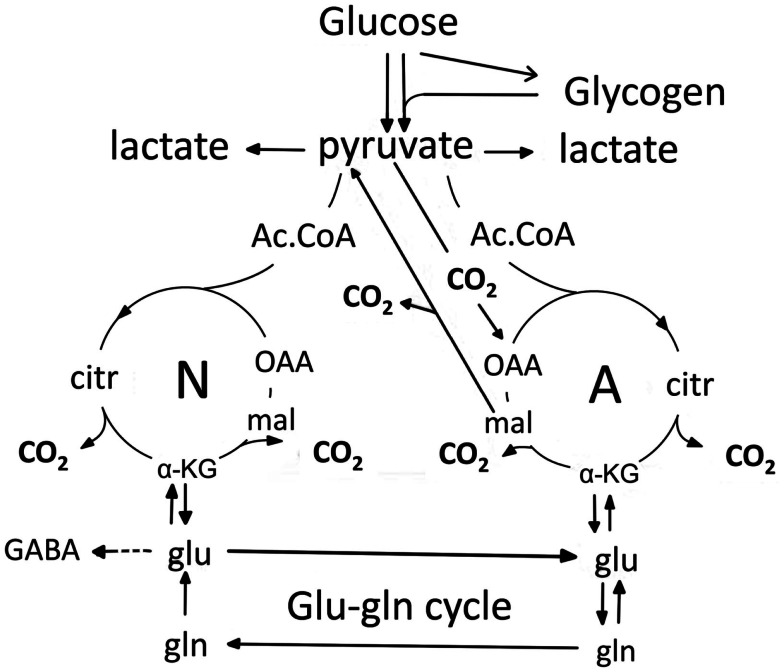
**Cartoon of glucose metabolism via pyruvate in neurons (left – N) and astrocytes (right – A) and of glutamine–glutamate (GABA) cycling**. In both cell types pyruvate metabolism via acetyl Coenzyme A (ac.CoA) leads to formation of citrate by condensation with *pre-existing oxaloacetate* (OAA) in the tricarboxylic acid (TCA), an end-result of the previous turn of the cycle. Citrate oxidation in the TCA cycle includes two decarboxylations, leading to re-formation of oxaloacetate, ready for another turn of the cycle, and to production of large amounts of energy (ATP). Pyruvate carboxylation creates a *new molecule* of oxaloacetate, which after condensation with acetyl Coenzyme A, derived from a second molecule of pyruvate, forms a new molecule of citrate. This process can be used for replacement of worn TCA cycle intermediates. More important in the present context is that α-ketoglutarate (α-KG), one of the intermediates of the TCA cycle can leave the cycle to form glutamate (glu) and, catalyzed by the cytosolic and astrocyte-specific enzyme glutamine synthetase, glutamine (gln). After release from astrocytes glutamine is accumulated in glutamatergic and GABAergic neurons [lower line (*V*_gln_ in the ^13^C-NMR studies) of the glutamine–glutamate (GABA) cycle (glu–gln cycle), converted to glutamate (and in GABAergic cells onward to GABA)] and released as transmitter. Released glutamate is almost quantitatively re-accumulated in astrocytes, together with part of the released GABA [upper line (*V*_cyc_ in the ^13^C-NMR studies) of the glutamine–glutamate (GABA) cycle (glu–gln cycle)] and re-accumulated in the astrocytic cytosol. Here, about 85% is converted to glutamine and re-enters the glutamine–glutamate (GABA) cycle. The remaining 15% is oxidatively degraded after re-conversion via α-ketoglutarate to malate, exit of malate to the cytosol, decarboxylation to pyruvate by the already mentioned cytosolic malic enzyme and further pyruvate oxidation in the TCA cycle via acetyl Coenzyme A. Combined astrocytic formation and oxidation of glutamate creates almost as much ATP as direct oxidation of glutamate (Hertz et al., 2007).

Figure 1 is a cartoon of selected parts of glucose metabolism in astrocytes (right) to neurons (left). They are connected by a flow of glutamine (produced directly from glutamate, generated as discussed below) from astrocytes to neurons. In the neurons glutamate is converted to transmitter glutamate and GABA. However, after their release as transmitters most glutamate and a considerable amount of GABA are returned to astrocytes. This is the glutamine–glutamate (GABA) cycle. Elegant ^13^C-NMR analysis (*in vivo* injection of labeled glucose, or in some cases acetate, and determination of labeled metabolites) has shown that the glutamine flux in the cycle, *V*_gln_ in the ^13^C-NMR studies, is slightly greater than the flux, *V*_cyc_ in the ^13^C-NMR studies, of released transmitter glutamate and GABA in the opposite direction (Rothman et al., 2011), and that GABA fluxes account for up to 20% of total flux in *V*_cyc_ (Patel et al., 2005; Chowdhury et al., 2007a). The reason for the slightly smaller *V*_cyc_ than *V*_gln_ may mainly be that some GABA is re-accumulated in GABAergic neurons (Schousboe, this Research Topic), where it can be oxidized (Yu, 1984). However, as discussed above, a considerable amount of GABA is also transferred to astrocytes, where it is taken up (Hertz et al., 1978a), transaminated to succinic acid semialdehyde (SSA), oxidized to succinate and then either (i) exits the tricarboxylic acid (TCA) cycle as malate (Figure 1); or (ii) is converted via α-ketoglutarate and glutamate to glutamine and returned to neurons in the glutamine–glutamate (GABA) cycle. Cytosolic malate is decarboxylated by the astrocyte-specific (Kurz et al., 1993), remarkably active (McKenna et al., 1995; Vogel et al., 1998) cytosolic malic enzyme to pyruvate, which can then be completely oxidized in the TCA cycle. Using this route, released glutamate is almost quantitatively taken up by astrocytes (Danbolt, 2001), and either converted to glutamine and reintroduced in the glutamine–glutamate (GABA) cycle, or metabolized to α-ketoglutarate by glutamate-dehydrogenase (GDH) or aspartate–glutamate transferase (AAT), followed by α-ketoglutarate oxidation after malate exit and decarboxylation. Glutamate oxidation is intense in cultured astrocytes (Yu et al., 1982; Hertz et al., 1988; McKenna, 2012), and increases with increasing glutamate concentration (McKenna et al., 1996). *In vivo* ∼85% of the accumulated glutamate is converted to glutamine and re-used, whereas the last 15% is oxidatively degraded (Rothman et al., 2011). *The close quantitative correlation between V_cyc_ and rate of glucose oxidation suggests that over 80% of neuronal oxidative ATP production is coupled to neuronal signaling even in the absence of specific stimulation* (Rothman et al., 2011).

The operation of glutamine–glutamate (GABA) cycle in one direction only is a result of the *astrocyte*-specific (probably *not* *glia-*specific) localizations of the enzymes pyruvate carboxylase, PC (Yu et al., 1983; Shank et al., 1985; Hutson et al., 2008) and glutamine synthetase, GS (Norenberg and Martinez-Hernandez, 1979; Derouiche, 2004). Pyruvate carboxylase is the enzyme catalyzing formation of oxaloacetate (OAA in Figure 1) from pyruvate. This is the only enzyme catalyzing net synthesis from glucose of a *new* TCA intermediate. Cytosolic malic enzyme normally only operates toward decarboxylation. The ubiquitously expressed pyruvate dehydrogenase (PDH) carries pyruvate, via pyruvate dehydrogenation and formation of acetyl Coenzyme A, into the TCA cycle in both neurons and astrocytes, but no new TCA cycle intermediate is generated by the action of this enzyme *alone*. This is because the citrate (citr), which is formed by condensation of acetyl Coenzyme A with pre-existing oxaloacetate in the TCA cycle loses two molecules of CO_2_ during the turn of the cycle, which leads to re-generation of oxaloacetate. This mechanism allows addition of another molecule of pyruvate in the next turn of the TCA cycle to continue the process, but it does not provide a *new* molecule of a TCA cycle intermediate that the cycle can afford to release and convert to glutamate. In contrast *joint* activity of PDH and PC activity creates a *new* molecule of citrate (Figure 1), which via α-ketoglutarate can be converted to glutamate, and by the aid of glutamine synthetase converted to glutamine. The pyruvate carboxylase is activated by enhanced brain function, as shown by an increase in CO_2_ fixation with brain activity in the awake rat brain (Öz et al., 2004). After termination of increased brain activity this effect may be reversed by increased glutamate degradation (see also below). Pyruvate can also be formed glycogenolytically from glycogen, previously generated from glucose (not shown), but glycogen turnover and glycogenolysis are slow processes (Watanabe and Passonneau, 1973; Dienel et al., 2002; Öz et al., 2012), except perhaps for occasional rapid bouts of glycogenolysis during very short time periods (Hertz et al., 2003). Glycogenolysis seems thus to be incapable of contributing much to metabolic fluxes, although blockade of glycogenolysis during sensory stimulation of awake rats does increase glucose utilization (Dienel et al., 2007). As discussed below, glycogenolysis seems mainly to serve as a fuel for signaling pathways, which are activated by stimulation of glycogenolysis, either by increased extracellular K^+^ concentrations (Hof et al., 1988) or transmitter effects (Magistretti, 1988; Subbarao and Hertz, 1990).

As illustrated in Table 1 the rate of flux in the glutamine–glutamate (GABA) cycle *in normal rat brain cortex* is only slightly lower than that of neuronal glucose oxidation (Sibson et al., 1998; Rothman et al., 2011, 2012; Hyder et al., 2013). Publications by these authors also show that the slight difference between the two fluxes is due to the persistence during deep anesthesia of a small amount of glucose oxidation but no glutamine–glutamate (GABA) cycling, whereas there is an approximately 1:1 ratio between the two parameters under all other conditions. This includes brain stimulation (Chhina et al., 2001; Patel et al., 2004).

**Table 1:** **Approximate metabolic rates in the non-anesthetized brain cortex from a multitude of ^13^C-NMR studies cited in text**.

Parameter	μmol/g wet wt per min
Rate of glucose oxidation	0.7
Rate of brain glucose utilization in astrocytes	20% of 0.7 = 0.14
Rate of glutamine–glutamate (GABA) cycle	0.6
Pyruvate carboxylase-mediated flux	50% of 0.14 = 0.07
Rate of glycogenolysis (Öz et al., 2012)	0.003

*Except for glycogenolysis, measured in humans, the values apply to the rat brain. For more details, see references cited in the text*.

**Figure 2:**
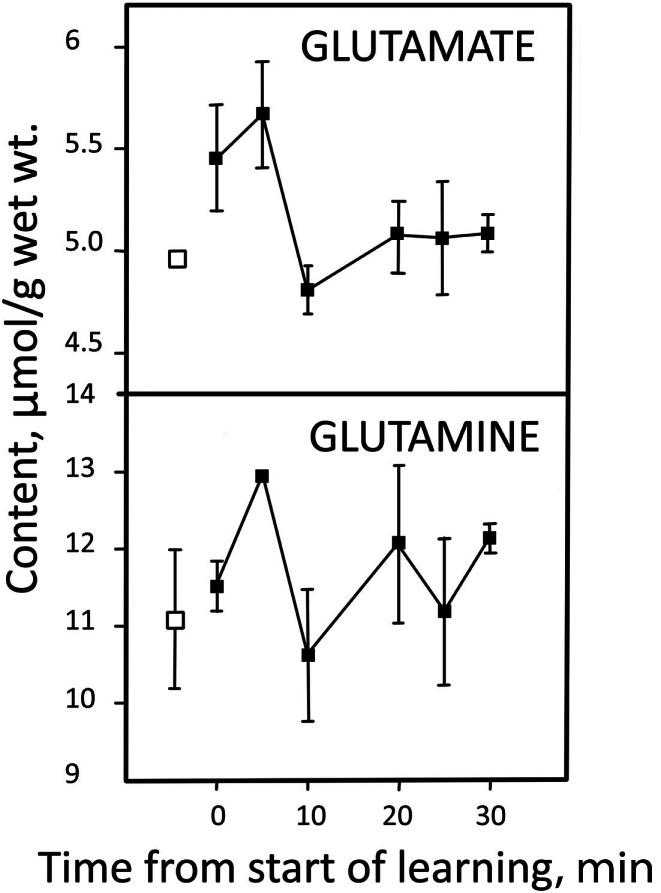
**Learning-induced changes in glutamate and glutamine content in the equivalent of the mammalian brain cortex in day-old chicken**. Pre-learning contents are indicated by open symbols and post-learning contents with filled-in symbols. From Hertz et al. (2003).

Stimulated brain activity is accompanied by a small immediate increase in glutamate content, associated with a quantitatively similar decrease in content of aspartate and with a slower decrease in content of glutamine (Dienel et al., 2002; Mangia et al., 2007; Lin et al., 2012). The matched increase in glutamate and decrease in aspartate may suggest an activity-induced alteration in relative distribution of these two amino acids in their association with the malate–aspartate shuttle (MAS) (Mangia et al., 2012). However, a larger increase in glutamate content without concomitant decrease in aspartate observed in an epileptic patient almost certainly represents increased *de novo* synthesis (Mangia et al., 2012). The same probably applies to a short-lasting increase in glutamate, together with a similar increase in glutamine (Figure 2) and aspartate (not shown) during learning (Hertz et al., 2003; Gibbs et al., 2007). The rapid subsequent return to normal amino acid levels is most likely brought about by enhanced degradation.

Oxidative metabolism in astrocytes is a *sine qua non-for* operation of the glutamine–glutamate (GABA) cycle. Pioneering studies early in this century (Gruetter et al., 2001; Lebon et al., 2002) showed that neurons account for up to 75% of oxidative glucose metabolism in the living brain and that astrocytes contribute most of the rest. These studies have been consistently and repeatedly confirmed in both human and rodent brain, and many of the rates are tabulated by Hertz (2011b). Since the volume occupied by astrocytes is similar to, or smaller, than the relative contribution of these cells to energy metabolism, their rate of oxidative metabolism per cell volume must be as high, if not higher, than that of neurons (Hertz, 2011b). This conclusion is consistent with an at least similarly high expression of most enzymes involved in oxidative metabolism of glucose in astrocytic as in neuronal cell fractions freshly obtained from the mouse brain (Lovatt et al., 2007).

## The Glucose-to-Glutamate Pathway

**Figure 3:**
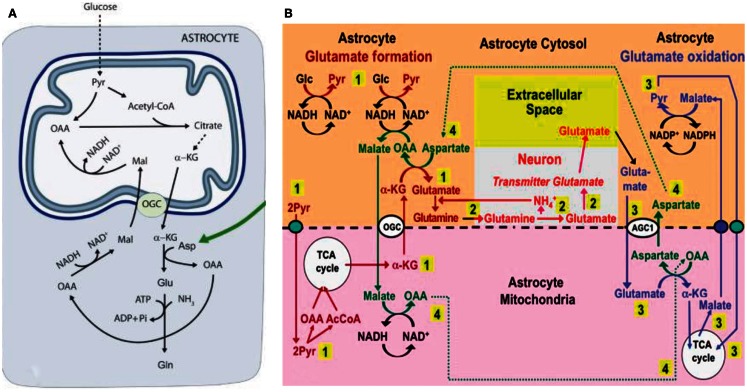
**(A)** Cartoon describing metabolic pathway from pyruvate to glutamate/glutamine in astrocytes, as suggested by Pardo et al. (2011). Joint pyruvate carboxylase and pyruvate dehydrogenase activation generates a “new” molecule of citrate as described above. Citrate-derived α-ketoglutarate exiting the mitochondrial membrane leaves the astrocytic TCA cycle and is transaminated with aspartate to form glutamate, with concomitant oxaloacetate (OAA) formation from aspartate. The mitochondrial exit of α-ketoglutarate occurs via the ketoglutarate/malate exchanger, generally acknowledged to be expressed in astrocytes, and the cytosolic malate with which is exchanged, is generated via NADH-supported reduction of aspartate-generated oxaloacetate. No aralar-requiring aspartate/glutamate exchanger (Slc1) activity is involved. **(B)** Proposed expansion by Hertz (2011a) of the model shown in **(A)**. The expanded model shows astrocytic production of glutamine (pathway 1), its transfer to glutamatergic neurons (without indication of any extracellular space, because there is no other function for extracellular glutamine than astrocyte-to-neuron transfer) and extracellular release as the transmitter glutamate (pathway 2), and subsequent reuptake of glutamate and oxidative metabolism in astrocytes (pathway 3), with connections between pathways 1 and 3 shown as pathway 4. Biosynthesis of glutamine is shown in brown and metabolic degradation of glutamate in blue. Redox shuttling and astrocytic release of glutamine and uptake of glutamate are shown in black, and neuronal uptake of glutamine, hydrolysis to glutamate, and its release is shown in red. Reactions involving or resulting from transamination between aspartate and oxaloacetate (OAA) are shown in green. Small blue oval is pyruvate carrier into mitochondria and small purple oval malate carrier out from mitochondria. AGC1, aspartate/glutamate exchanger, aralar; α-KG, α-ketoglutarate; Glc, glucose; Pyr, pyruvate; OGC, malate/α-ketoglutarate exchanger. It should be noted that (i) aralar activity is required initially for reversal of cytosolic NAD^+^/NADH changes occurring during the one oxidative process occurring during pyruvate formation, but subsequently not in astrocytes until the oxidation of glutamate, probably allowing rapid glutamate synthesis, and (ii) all reactions are stoichiometrically accounted for **(A**) From Pardo et al. (2011); **(B)** From Hertz (2011a).

In cultured cerebellar astrocytes conversion of glutamate to α-ketoglutarate at least mainly occurs via a transamination (Westergaard et al., 1996). This is consistent with a recent *in vivo* study by Pardo et al. (2011), which established that the contents of glutamate and glutamine in cultured astrocytes increase by ∼50% in the presence of aspartate at a concentration of ≥100 μM, but not in the presence of alanine or leucine. On the basis of this finding the authors suggested the pathway shown in Figure 3A, according to which glucose-derived α-ketoglutarate leaves the astrocytic TCA cycle in exchange with malate, generated via oxaloacetate (OAA), which in turn had been formed from aspartate *in a transamination process*. Subsequently OAA is reduced to malate (MAL), with concomitant oxidation of NADH to NAD^+^. The entire process requires operation of the α-ketoglutarate/malate exchanger (OGC in Figure 3A), but not of the aspartate/glutamate exchanger AGC, in brain AGC1. On the basis of their own and previous immunocytochemical observations in brain tissue by themselves and others (Ramos et al., 2003; Berkich et al., 2007), Pardo et al. (2011) regarded this exchanger as absent or sparsely expressed in astrocytes because of deficient expression of aralar, a necessary component of AGC1.

**Figure 4:**
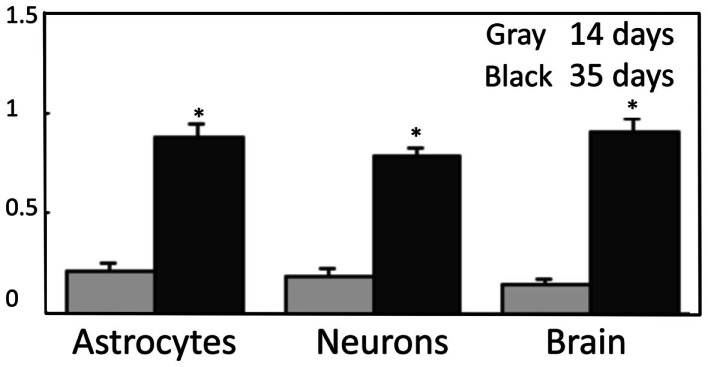
**Protein expression of aralar in neuronal and astrocytic cell fractions are similar and develop at identical rates**. Neuronal and astrocytic cell fractions were gently isolated from two mouse strains, one expressing a neuronal marker with a specific fluorescence and the second expressing an astrocytic fluorescent signal (Lovatt et al., 2007). Samples were applied to slab gels of 12% polyacrylamide, separated by electrophoresis, and transferred to nitrocellulose membranes. After blocking with 5% skim milk powder and washing, these membranes were incubated for 2 h at room temperature with the primary anti-aralar antibody sc-271056 (from Santa Cruz Biotechnology, CA, USA) after dilution (1 × 100), shown not to react with the related carrier citrin, and followed by incubation with a goat anti-mouse HRP-conjugated secondary antibody, also from Santa Cruz Biotechnology (dilution: 1 × 200) for 2 h at room temperature. Anti β-actin (Sigma, St. Louis, MO, USA) was applied in the same lanes as the aralar antibody to use β-actin as a housekeeping protein providing an internal control for protein load. The intensities of both bands on the blots were scanned, and the ratios between aralar and β-actin were calculated and shown in the Figure. From Li et al. (2012b).

Subsequently Hertz (2011a), suggested that (i) the reduction of oxaloacetate to malate was a necessary compensatory consequence of the reduction of NAD^+^ to NADH during the one oxidative process during glycolysis (glyceraldehyde-3-phosphate to 1-3-biphosphoglycerate), without which normally no production of α-ketoglutarate can occur from glucose, and (ii) that aspartate, *formed from OAA in astrocytes when glutamate during its oxidation is transaminated to α-ketoglutarate*, *supplied the needed aspartate*, as illustrated in Figure 3B. The latter suggestion required exit to the cytosol of mitochondrially located aspartate via the aralar-dependent AGC1 in the MAS. The involvement of the MAS during glutamate oxidation, but not during its synthesis (Figure 3A) might contribute to the development of MAS-based alteration in glutamate/aspartate ratio during brain activation suggested by Mangia et al. (2012). The suggestion of malate–aspartate participation in Figure 3B was felt to be justified by the finding by Lovatt et al. (2007) of equal expression of mRNA for aralar, determined by microarray analysis, in freshly isolated astrocytes and neurons. Moreover, it was calculated (based on data by Berkich et al., 2007) that the aralar expression found by Pardo et al. (2011) sufficed to produce enough aralar for the proposed model to function, although malate–aspartate cycle activity needed for synthesis of α-ketoglutarate at the beginning of the process increase demands. Equally high levels of mRNA aralar expression is astrocytes were later confirmed, *and its protein expression* (Figure 4) shown in freshly separated astrocytes and neurons from isolated cell fractions (Li et al., 2012b). The separation procedure used selects astrocytes indiscriminately, but among neurons it mainly isolates glutamatergic projection neurons. These experiments also demonstrated *remarkably large differences in aralar expression in young and mature animals*. This finding was replicated in cultured astrocytes, whereas homogeneous neuronal cultures are too short-lived to provide meaningful results.

The model suggested in Figure 3B is consistent with the important ^13^C labeling data in the study by Pardo et al. (2011) in young aralar^−/−^ animals, showing incorporation of [^13^C]glucose into glutamate but not into glutamine. This is because the absence of aralar does not exclude mitochondrial glutamate synthesis, especially if a substantial amount of α-ketoglutarate is supplied from non-glucose source in these young animals. However, in aralar^−/−^ animals de-amidation of glutamine *in neurons* may be impaired as will be discussed below, which may prevent glutamine synthesis in astrocytes.

Formation of glutamate from glucose requires glycogenolysis, both in the intact chicken brain (Gibbs et al., 2007) and in cultured astrocytes (Sickmann et al., 2009). Absence of glycogen phosphorylase in oligodendrocytes (Richter et al., 1996) therefore is a powerful argument against functioning pyruvate carboxylase activity in oligodendrocytes. The rate of glycogenolysis in brain (Table 1) is not high enough that pyruvate derived from glycogen could be used by the astrocytes as the sole source of pyruvate for carboxylation. Most, although probably not all glucose oxidation in astrocytes proceeds via glutamate formation, astrocytes account for 20% of total glucose oxidation rate, or 0.14 μmol/g per min (in the rat), and one half of glutamate formation (0.07 μmol/g per min) occurs *via* pyruvate carboxylation (with the other one half mediated by PDH). This exceeds the rate of glycogenolysis by at least 10 times. Rather, as in the case of other astrocytic processes requiring activation of specific signaling pathways (Xu et al., 2013), glycogenolysis seems to be required for signaling processes needed to activate pyruvate carboxylase activity. Glycogenolysis is stimulated by even very small increases in extracellular K^+^ concentrations above their normal level (Hof et al., 1988), and in astrocyte cultures pyruvate carboxylation is increased by an elevation of the K^+^ concentration in the medium (Kaufman and Driscoll, 1993). Pyruvate carboxylation at least in other cell types (Garrison and Borland, 1979) is also stimulated by noradrenaline, as is astrocytic glycogenolysis (Magistretti, 1988; Subbarao and Hertz, 1990). This does not mean that a very brief increase in glutamate content, as shown in Figure 2 might not, at least partly, be derived from glycogen, which showed a simultaneous precipitous and large fall (Hertz et al., 2003).

Formation of glutamine from glutamate in the astrocytic cytosol is in agreement with the astrocyte-specific expression of glutamine synthetase (Norenberg and Martinez-Hernandez, 1979), with probable lack of expression in oligodendrocytes confirmed by Derouiche (2004). In cultured astrocytes reduced function of the glutamine synthetase after administration of its inhibitor, methionine sulfoximine (MSO), causes an increase in glutamate and aspartate formation, the latter probably reflecting increased glutamate oxidation, when glutamine synthesis is inhibited (Zwingmann et al., 1998). Increased content of aspartate in brain slices during MSO inhibition has also been shown by Nicklas (1983). Aspartate production by this route might under adverse conditions supplement the aspartate needed for transamination, when α-ketoglutarate is converted to glutamate (Figures 3A,B). Chronic infusion of MSO into rat hippocampus increases glutamate content, specifically in astrocytes, by almost 50%, but has remarkably little effect on glutamate in synaptic endings (Perez et al., 2012). The animals develop seizures, and the authors suggested that the extracellular brain glutamate concentration had become increased, perhaps due to excessive release of glutamate and/or decreased extracellular clearance.

Glutamine can travel between gap-coupled astrocytes, and the distance it reaches increases during brain activation (Cruz et al., 2007). Different transporters have been proposed to *direct* its transport from astrocytes to neurons, but it now appears well established that glutamine release occurs via the amino acid transporter SN1. This transporter is densely expressed in astrocytic processes abutting glutamatergic and GABAergic neurons (Boulland et al., 2002). Efflux through SN1 is increased by acidic extracellular pH and by increased intracellular Na^+^ concentrations (Bröer et al., 2002). Uptake of Na^+^ in astrocytes during re-accumulation of excess extracellular K^+^ from the extracellular space after neuronal excitation (Xu et al., 2013) might therefore increase glutamine release. Extracellular glutamine is taken up into neurons by SAT1,2 (Kanamori and Ross, 2006; Blot et al., 2009; Jenstad et al., 2009). This topic is discussed in detail in the paper by Chaudhry et al. in this Research Topic.

**Figure 5:**
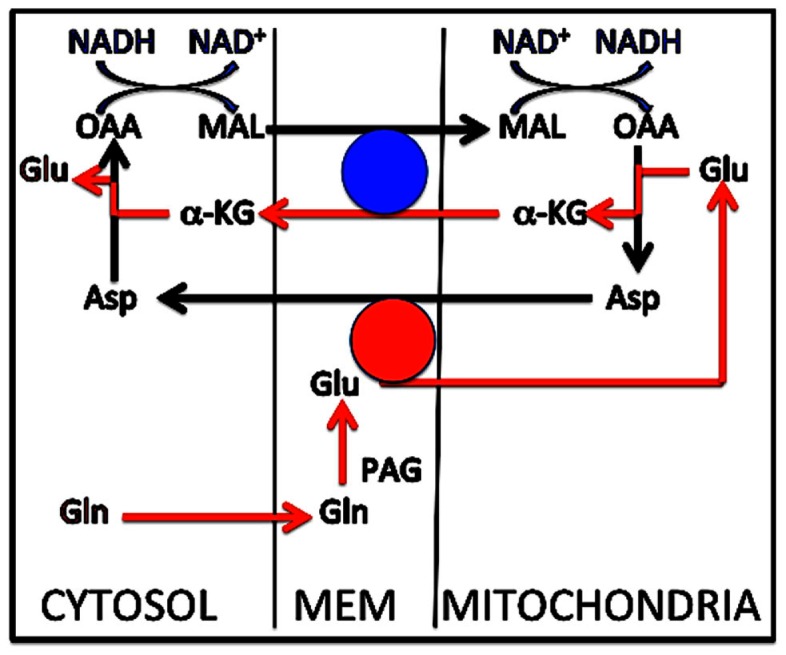
**Metabolic pathway for conversion of glutamine to glutamate in cultured cerebellar granule neurons**. Glutamine enters the intermembranaceus space from the cytosol (red arrow at bottom of Figure). Here glutamate is formed by phosphate-activated glutaminase (PAG), but instead of returning to the cytosol, it enters the mitochondrial lumen and is transaminated to aspartate, coupled with transamination of oxaloacetate (OAA) to α-ketoglutarate (α-KG). As in the malate–aspartate shuttle α-KG exits the mitochondrial membrane in exchange with incoming malate, and intramitochondrial malate is oxidized to oxaloacetate. The mitochondrially generated aspartate is the source of the cytosolic malate exchanging with α-KG after it has been transaminated to oxaloacetate and reduced to malate. Two transmitochondrial carriers are involved, (1) the glutamate/aspartate exchanger AGC1, shown by a red circle, which requires aralar [undisputed presence in neurons, except in the aralar^−/−^ mice studied by Pardo et al. (2011), where glutamate synthesis may have been abrogated], and (2) the α-ketoglutarate/malate carrier (OGC), shown by a blue circle. As mentioned in the text this process is similar to that operating in the malate–aspartate shuttle, with the exception that glutamate in the latter originates in the cytosol, not in the mitochondrial intermembranaceus space. The interactions between oxaloacetate and malate must be coupled to conversion of NADH to NAD^+^ in the cytosol and the reverse change in the mitochondria (top of Figure). This contributes to the quantitative correlation between glucose flux and glutamine/glutamate (GABA) cycle activity at different activity ranges. Modified from Palaiologos et al. (1988).

Although not shown in Figure 3B (for the sake of simplicity), the subsequent de-amidation of glutamine to glutamate appears to be somewhat complex, probably reflecting the subcellular localization of the phosphate-activated glutaminase (PAG). In cultured glutamatergic neurons inhibitor studies have suggested the pathway indicated in Figure 5 (Palaiologos et al., 1988). This Figure shows conversion of glutamine to glutamate by PAG, followed by a process similar to that occurring in the MAS, with the only exception that the glutamate molecule involved does not originate in the cytosol, but from PAG-activated de-amidation of glutamine in the intermembranaceus space of the mitochondrion. This mechanism implies a concomitant mitochondrial reduction of NAD^+^ to NADH, associated with malate oxidation to OAA, cytosolic oxidation of NADH to NAD^+^, and reduction of oxaloacetate to malate. Evidence that a similar process occurs in freshly isolated mitochondria (Bak et al., 2008) and description of perhaps even more complicated processes in GABAergic neurons are discussed by Schousboe et al. in the present Research Topic.

${\text{NH}}_{4}^{+}$ released during the glutaminase reaction (and/or the small amount of NH_3_ present at physiological pH) may easily traverse the outer, permeable mitochondrial membrane to reach the neuronal cytosol. A large number of studies have attempted to investigate ammonia transport from here to the astrocytic cytosol, where it is needed for continuous glutamine production. Many of these have focused on potential amino acid shuttles, capable of mediating this transport, but a recent review by Rothman et al. (2012) has shown too little transport capacity of these cycles in the brain *in vivo* to be entirely responsible for this function. This does not mean that they could not have a back-up function. This conclusion may re-focus attention on channel- and/or transporter-mediated contributions to efflux from neurons and influx into astrocytes (Benjamin, 1987). A major ion extruder in neurons is the K^+^–Cl^−^ co-transporter KCC2 (Chamma et al., 2012; Löscher et al., 2013), and Marcaggi and Coles (2000) has shown rapid ammonia exit from neurons in the bee retina via a co-transporter. Fittingly, in cultured astrocytes Nagaraja and Brookes (1998) showed that channel- and NKCC1-mediated ${\text{NH}}_{4}^{+}$ uptake together accounted for an uptake, which was similar in magnitude to the glutamate uptake in similar cultures. At 1 mM extracellular NH_4_Cl it amounted to 30 nmol/mg protein per min, which *in vivo* (100 g protein/g wet wt.) would equal 3 μmol/g wet wt per min, or about one half of the *in vivo* rate of the glutamine–glutamate (GABA) cycle (Table 1). At the same time the cytosol became acidic. Reversal of intracellular acidosis by NHE1 and NBCe1 acid extruders (Song et al., 2008, 2012) would create extracellular acidosis, stimulating SN1-mediated glutamine release. Na^+^,K^+^-ATPase activity is required both to support NKCC1 function, since NKCC1 operates as a secondary active transporter supported by Na^+^,K^+^-ATPase-generated ion gradients, and to maintain conditions allowing inward channel-mediated ${\text{NH}}_{4}^{+}$ transport. Inhibition of glutamine formation and retention in rat brain slices by ouabain (Benjamin, 1987) might therefore result from impairment of channel and transporter activity.

Regardless of detailed mechanisms involved, *ATP requirement for glutamine synthesis and ammonia uptake in astrocytes and for glutamine (or GABA) uptake in neurons makes neuronal transmitter supply from astrocytes an approximately two–three times more expensive process than neuronal production or uptake from the extracellular fluid would have been*.

## Return and Re-Use of Released Glutamate

**Figure 6:**
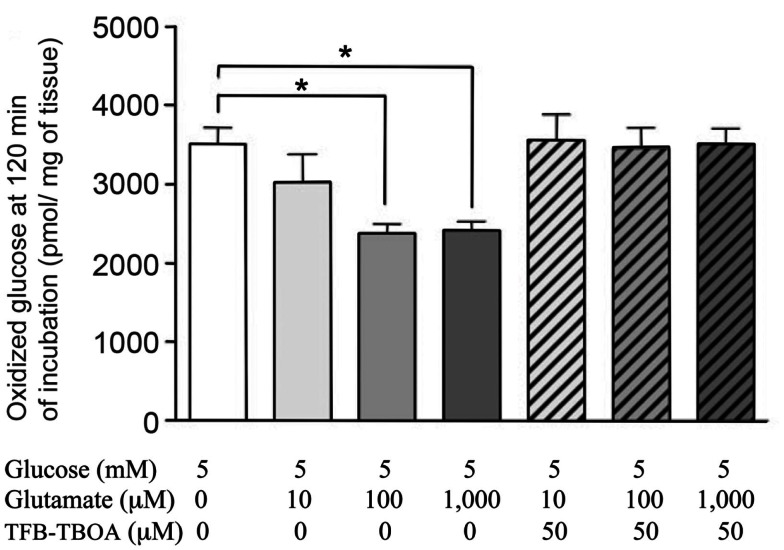
**Glucose oxidation rates of intact rat hippocampi, incubated in tissue culture medium for 2 h with [U-^14^C] glucose**. From Torres et al. (2013).

It is well established that virtually all released transmitter glutamate is accumulated specifically into astrocytes by the two transporters GLAST (EAAT1) and Glt1 (EAAT2) (Danbolt, 2001). Claims to the contrary can generally be discounted as due to artifacts, e.g., homo- or hetero-exchange with intracellular amino acids. There is also no doubt that intact brain tissue can oxidize glutamate. Brain slices show higher respiratory activity during incubation in a medium containing l-glutamate as the only substrate than in the absence of any substrate (Dickens and Greville, 1935; Abadom and Scholefield, 1962). Nevertheless, according to most authors (Lipsett and Crescitelli, 1950; Ghosh and Quastel, 1954) rodent brain slices incubated with l-glutamate show a lower rate of oxygen consumption than during incubation with glucose alone. These findings are consistent with glutamate being a metabolic substrate that can be utilized by some, but not all cells, in the tissue. A recently demonstrated ability of glutamate to decrease the rate of glucose oxidation in incubated rat hippocampi (Torres et al., 2013) is reproduced in Figure 6 and shows that oxidation of glutamate can supply energy supporting not only the increased demand created by its own uptake (e.g., McKenna, 2012), but also demands normally fueled by glucose oxidation. The relative low rates of apparent glucose oxidation in such experiments is well known, partly due to the incubation *in vitro* and partly to the late turns of the TCA cycle during which some the labeled atoms are oxidized. Although it may be doubtful if glutamate oxidation is *necessary* metabolically, it may be functionally very important for complete disposal of glutamate in the brain *in vivo*, with continuous glutamate *de novo* synthesis and degradation and paucity/absence of other disposal or dilution routes.

In astrocyte cultures (Eriksson et al., 1995; Hertz and Hertz, 2003) l-glutamate is an excellent substrate for oxidative metabolism. The observation by Yu et al. (1982) that glutamate oxidation is insensitive to the transamination inhibitor AOAA and therefore initiated by GDH activity has been confirmed by virtually all authors with the exception of Hutson et al. (1998). However, this *in vitro* observation may not be valid for the brain *in vivo*, and in glutamate-dehydrogenase knock-out mice glutamate oxidation, determined in astrocyte cultures is reduced, but absolutely not abolished (Frigerio et al., 2012). Moreover, glutamate-dehydrogenase-mediated glutamate oxidation disagrees with the observation by Balazs (1965) that in brain mitochondria by far most of the glutamate conversion to α-ketoglutarate is catalyzed by the aspartate aminotransferase, an observation confirmed in both synaptic and non-synaptic mitochondria by Berkich et al. (2007). These observations raise the question whether lack of some of the many mechanisms regulating GDH activity (McKenna, 2011; Li et al., 2012a) may not function in isolated mitochondria, or whether use of isolated astrocytes without the possibility of metabolic interactions between glutamate synthesis ad glutamate oxidation may have caused the GDH dependency (see also below). Observations by Wysmyk-Cybula et al. (1991) in freshly isolated cerebral astrocytes and by Rao and Murthy (1993) in isolated cerebellar astrocytes that glutamate oxidation is mainly transaminase-dependent, support the latter conclusion. Additional support may be provided by the stimulation of glutamate *production* by aspartate shown by Pardo et al. (2011), since transactivation during glutamate *oxidation* can provide the needed aspartate (Figure 3B), but GDH-mediated α-ketoglutarate cannot. In contrast to glutamate oxidation via GDH, that by the aspartate transaminase also requires use of oxaloacetate, in the proposed model generated during glutamate production (labeled 4 close to the lower edge of Figure 3B).

Patients suffering from temporal lobe epilepsy, with sclerotic astrocytes and neuronal loss, often display a combination of highly elevated extracellular glutamate concentrations, also interictally, interictal hypometabolism, reduced glutamine synthetase activity and greatly reduced glutamine formation from glutamate (Petroff et al., 2002a,b; Eid et al., 2012). As during chronic perfusion of normal animals with MSO (Perez et al., 2012), the interictal elevated glutamate may largely reflect higher cellular glutamate levels within astrocytes, probably due to chronic impaired glutamine synthetase function and perhaps also impaired oxidation, since neuronal number and volume is reduced much more than total tissue glutamate levels (D. L. Rothman, personal communication). It would be interesting actually to determine extracellular glutamate concentration in the animals treated by Perez et al. (2012), since Exposito et al. (1994) previously showed that *acute* intrastriatal administration of MSO decreases the extracellular glutamate concentration.

Glutamate synthesis and degradation in differently located astrocytes was envisaged to present a possible problem in the suggested interacting pathways of glutamate production and oxidation with its exclusive use of aspartate aminotransferase (Figure 3B) rather than GDH (Hertz, 2011a). It was discussed that trans-astrocytic transport of co-factors and metabolites and lactate formation may alleviate this problem, but this may not always be sufficient, and lack of GABA return, and thus oxidation, in astrocytes will aggravate the situation. Pronounced expression in non-synaptic mitochondria of both AOAA- and non-AOAA-sensitive glutamate oxidation pathways (Table 2) may suggest a back-up function of the GDH. This would be consistent with the findings by Balazs (1965) and Wysmyk-Cybula et al. (1991) that transaminase-dependent glutamate oxidation accounted for most, but not all, glutamate oxidation. However, in GDH 1 knock-out mice (only humans express GDH 2) most functions, except the already mentioned reduced glutamate oxidation in cultured astrocytes, are remarkably intact (Frigerio et al., 2012). This applies to synaptic activity and long-term potentiation (LTP), an observation which may exclude that GDH in intact tissue should be essential for glutamate oxidation in astrocytes. However, glutaminase activity, glutamine content, and expression of the two astrocytic transporters Glt1 and GLAST were moderately increased in the knock-out animals. These alterations are consistent with, but do not prove, that glutamate production is rendered slightly more difficult, but certainly not abolished, when GDH is silenced.

**Table 2:** **Oxygen consumption rates as indications of kinetics of glutamate-dehydrogenase and aspartate–glutamate transferase activities in non-synaptic mitochondria from the rat**.

	High-affinity Km (mM)	High-affinity *V*max (μmol O_2_/min per g)	Low-affinity Km (mM)	Low-affinity *V*max (μmol O_2_/min per g)
Glutamate + malate	0.26	3.75	1.3	7.65
Glutamate + malate + AOAA	0.19	2.45		

*From Lai and Clark (1976)*.

Based on the sum of cited evidence it appears reasonable to suggest initiation of glutamate oxidation by transamination as the default pathway, but participation of GDH may occur in situations when an insufficient match between glutamate biosynthesis and degradation is not possible because of different rates, too long distances between biosynthesis and degradation sites, or prevailing conditions *in vitro*. The ability of the transaminase-mediated pathway to provide aspartate for glutamate synthesis is a strong argument for its function, and the increased aspartate content in brain slices after administration of MSO found by Perez et al. (2012) might partly reflect a decreased glutamate oxidation. In addition to the pathway suggested in Figure 3A, Hutson et al. (1998) has proposed a different pathway for aspartate transaminase-mediated glutamate oxidation. This model relies on many complex interactions, including branched chain amino acid cycling, but Rothman et al. (2012) concluded that it would be able to function in the mammalian brain *in vivo*, since *in vivo* data showed high enough fluxes and enzyme expression levels for this to be possible.

The total of four conversions of NAD^+^ to NADH in the joint glutamate synthesis/oxidation process suggested [two in astrocytes during the production of α-ketoglutarate and one during glutamate oxidation (Figure 3B), together with one in neurons (Figure 5)] may explain the close to 1:1 ratio between rates of glucose oxidation and of glutamine/glutamate (GABA) cycle flux. The repeated NAD^+^/NADH conversions also suggest much more complicated and repeated oxidation/reduction responses in brain cortex during neuronal and astrocytic activities than previously considered (e.g., Kasischke et al., 2004). It was also mentioned above that *over 80% of neuronal oxidative ATP production is coupled to neuronal signaling even in the absence of specific stimulation* (Rothman et al., 2011). What is peculiar, though, is that three of the four NAD^+^/NADH conversions occur in astrocytes. The initial formations of α-ketoglutarate from glucose do require malate–aspartate cycle activity, but there seem to be agreement that only 15–20% of glutamine–glutamate (GABA) cycle activity is connected with *de novo* synthesis of glutamate. Could glutamate destined for oxidation and for glutamine synthesis be segregated, and the pathways suggested in Figure 3A apply only to the former? This question makes it so important to determine the pathway(s) for glutamate degradation not only in isolated astrocytes but also in intact brain tissue (see papers by McKenna and by Whitelaw and Robinson in this Research Topic). Would studies of metabolism of labeled glutamate (with appropriate receptor antagonists) at least in brain slices be useful? Also, could glutamate conversion to α-ketoglutarate be catalyzed by aspartate aminotransferase in one potential subfraction and by GDH in the other? These are critical questions. Finally, even if virtually all glutamate is oxidized in astrocytes, pyruvate formed from malate could be converted to lactate and transferred to neurons, but most evidence does not support lactate transfer from astrocytes to neurons. Nevertheless, astrocytes do contribute energetically to glutamine, glutamate, and GABA homeostasis (by uptake and glutamine synthesis) to a similar degree as neurons (cellular uptake and vesicular accumulation of glutamate and GABA). The same is the case for clearance of excess extracellular K^+^ (astrocytic uptake followed by release and neuronal uptake after extracellular K^+^ clearance, and they seem even to be responsible for the post-excitatory undershoot in extracellular K^+^ concentration (Hertz et al., 2013). Since glutamate is the major excitatory transmitter, its release will cause efflux of neuronal K^+^, followed by extracellular K^+^ clearance and, after extensive stimulation, also post-excitatory undershoot in extracellular K^+^ concentration. Thus, glutamate-mediated, K^+^-associated excitatory activity may increase astrocytic energy demands as much as neuronal. One may wonder (i) if this dual uptake (and thus double metabolic billing), apparently of both glutamate and K^+^ is the major reason for the extremely high energy metabolism in brain, and (ii) whether signaling possibilities especially exist during transport through the astrocytic syncytium.

## Return and Re-Use of Released GABA

**Figure 7:**
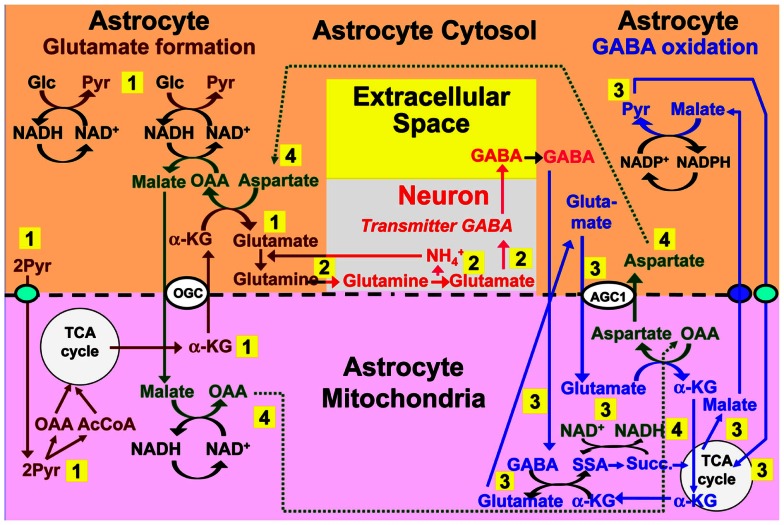
**Proposed model for cytosolic–mitochondrial trafficking associated with astrocytic production of glutamine (pathway 1), its transfer to GABAergic neurons (without indication of any extracellular space, because astrocyte-to-neuron transfer is the only major function for extracellular glutamine), neuronal GABA formation via glutamate (without details), and extracellular release as transmitter GABA (pathway 2), with subsequent reuptake of GABA and oxidative metabolism in astrocytes (pathway 3), and connections between pathways 1 and 3 shown as pathway 4**. Biosynthesis of glutamine is shown in brown and metabolic degradation of GABA in blue. GABA is suggested to enter the mitochondria in exchange with glutamate, although no such exchanger is presently known, and subsequently be transaminated to succinic-semialdehyde and oxidized to succinate. α-KG is initially used for the transamination, but later re-generated during metabolism of glutamate entering the mitochondria and metabolized as previously suggested for transmitter glutamate, with the mitochondrial malate extruder shown as a small purple oval. Redox shuttling and astrocytic release of glutamine and uptake of GABA are shown in black, and neuronal uptake of glutamine, followed by GABA formation and release in red. Reactions involving or resulting from transamination between aspartate and oxaloacetate (OAA) are shown in green. Note that (i) aralar activity is required not only for the initial production of each of two molecules of pyruvate (light blue oval in pathway 1), but also during the re-entry of glutamate into the TCA cycle and for the final entry of pyruvate into the mitochondria (light blue oval in pathway 3), (ii) all reactions are stoichiometrically accounted for, although only when GABA synthesis and oxidation are integrated. AGC1, aspartate/glutamate exchanger, aralar; α-KG, α-ketoglutarate; Glc, glucose; Pyr, pyruvate; OGC, malate/α-ketoglutarate exchanger.

Although GABA contributes at most 10–20% of fluxes in the glutamine–glutamate (GABA) shuttle, GABAergic signaling is essential in the regulation of endocrine functions and in brain information processing. It is therefore an important question whether oxidation of GABA in astrocytes might also be associated with the biosynthetic pathway. Pathways for such a potential interaction are suggested in Figure 7. The synthetic pathway is identical to that for glutamate up till GABA formation from glutamate, which is discussed by Schousboe et al. in this Research Topic. During metabolism the most uncertain point is how GABA enters the mitochondrion. The model suggests an exchange with glutamate, but no mitochondrial GABA/glutamate exchanger is known in mammalian cells, although an exchanger has been demonstrated in plants (Michaeli et al., 2011), and the cell membrane of prokaryotes (which have no mitochondria) express a glutamate–GABA exchanger, GadC (Ma et al., 2012). After its mitochondrial exit cytosolic glutamate follows a similar pathway as during glutamate oxidation. The relatively low content of glutamate in astrocytes (Storm-Mathisen and Ottersen, 1983; Storm-Mathisen et al., 1992), and thus also in astrocytic mitochondria, will not be affected, because mitochondrial glutamate is re-established via MAS-mediated uptake, initial transamination to α-ketoglutarate, coupled to transamination of oxaloacetate, and a second transamination coupled to GABA transaminase-mediated formation of SSA. The steps of GABA transaminations are conventional, as is its subsequent complete oxidation via succinate, malate, and pyruvate.

An increase in aspartate but a decrease in glutamate and glutamine contents and synthesis has been observed in brain cortex from 17-day-old succinic-semialdehyde dehydrogenase-deficient mice together with a pronounced decrease (40%) in glutamine synthetase expression, whereas GABA production was virtually unaffected (Chowdhury et al., 2007b). From Figures 3A,B follows that reduction of glutamate production, including initial transamination from α-ketoglutarate might explain both the increase in aspartate content and the reduced glutamine synthetase expression. On the other hand deficient GABA transamination should decrease aspartate production in the return route. Although degradation-synthesis coupling would be consistent with Figures 3A,B, it is also unexplained why metabolism of GABA should be so important for glutamate production, since GABA in adult animals contributes so relatively little to the return flux toward astrocytes (*V*_cyc_). However, by necessity 17-day-old mice were used (the gene-deficient animals die around day 21). As will be discussed below, the glutamine–glutamate (GABA) cycle is not fully developed in these immature animals. Moreover, in rat brain slices both GABA and glutamate uptake and release rates show very rapid and pronounced quantitative fluctuations during early development (Schousboe et al., 1976), and GABA fluxes may have been considerable in 16-day-old animals.

Metabolism of GABA along the pathway suggested in Figure 7 would eliminate a need for uptake of exogenous aspartate and its conversion to malate in astrocytes in an aspartate-dependent pathway model for GABA formation suggested by LaNoue et al. (2007). Such a pathway is not likely to operate, since aspartate itself must be synthesized in an astrocyte–neuron metabolic co-operation. Also, in contrast to glutamate, aspartate cannot sustain its own uptake in cultured astrocytes by oxidative metabolism (Peng et al., 2001), as it should have been able to do in order for the subsequent oxaloacetate-to-malate reduction suggested by LaNoue et al. (2007) to occur. These models were based on the assumption that astrocytes express no aralar. The repeated findings of aralar mRNA together with the additional demonstration of its protein expression in freshly isolated astrocytes probably mean that they can now be regarded as outdated.

## How Does the Brain Manage before the Development of the Glutamine–Glutamate (GABA) Cycle?

**Figure 8:**
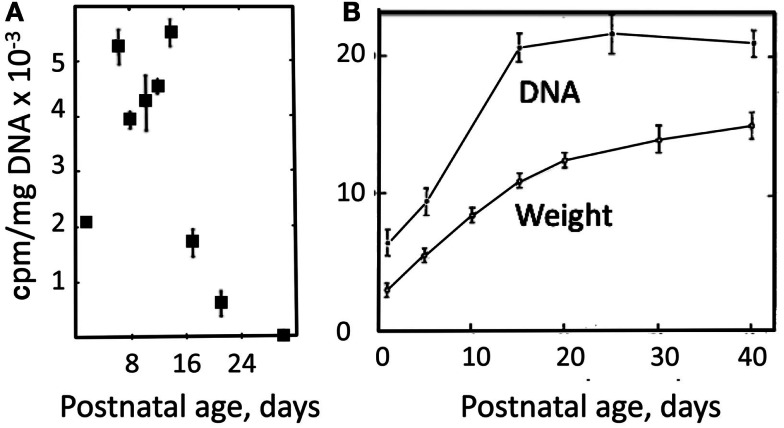
**(A)** Rate of DNA synthesis, measured in unincubated brain slices by incorporation ^14^C after [^14^C]thymidine exposure 12 h earlier. From Schousboe (1972). **(B)** Developmental increase in weight and DNA content of rat brain. From Mori et al. (1970).

The strikingly slow development of full aralar expression in rat brain and isolated brain cells (Figure 4) probably mainly reflects that gliogenesis in the rodent cerebral cortex is mainly postnatal (Altman, 1969b). A glial cell population, predominantly containing astrocytes, expands in the rodent brain cortex during the first 3 weeks of postnatal development, largely by local division (Ge et al., 2012). This is in contrast to a virtually completed neurogenesis at birth (Altman, 1969a; Bhardwaj et al., 2006). Mori et al. (1970) and Schousboe (1972) took advantage of the developmental difference between neurogenesis and gliogenesis to regard measured cell division in brain cortical tissue from postnatal day 6 and onward (by incorporation of label from [^14^C]thymidine) as mainly reflecting formation of astrocytes. This appears justified, since simultaneous proliferation of oligodendrocytes and vascular cells probably contribute less to total volume in gray matter than astrocytes and may be less condensed time-wise. Both groups found similarly intense and virtually constant cell proliferation rate for slightly more than one subsequent week, followed by its abrupt termination around postnatal day 15 (Figure 8A). The cell proliferation is accompanied by huge increases in brain weight and total DNA, also stopping around day 14 (Figure 8B).

**Figure 9:**
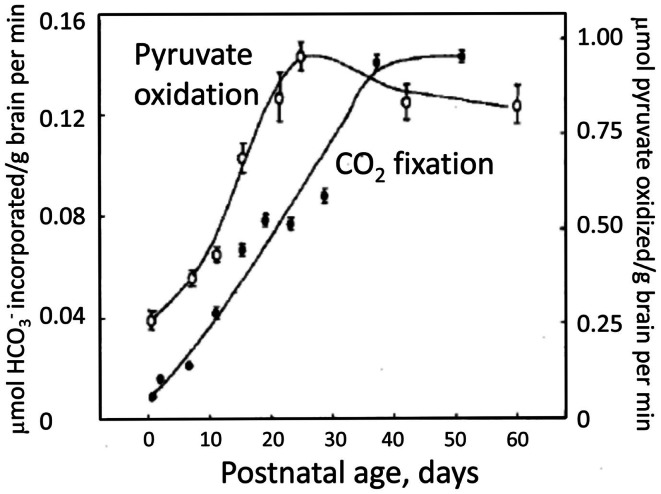
**Decarboxylation of [l-14C]pyruvate by pyruvate dehydrogenase and the fixation of ${\text{HCO}}_{3}^{-}$ by pyruvate carboxylase in rat brain homogenates obtained from animals of different ages between birth and adulthood**. From Wilbur and Patel (1974).

**Figure 10:**
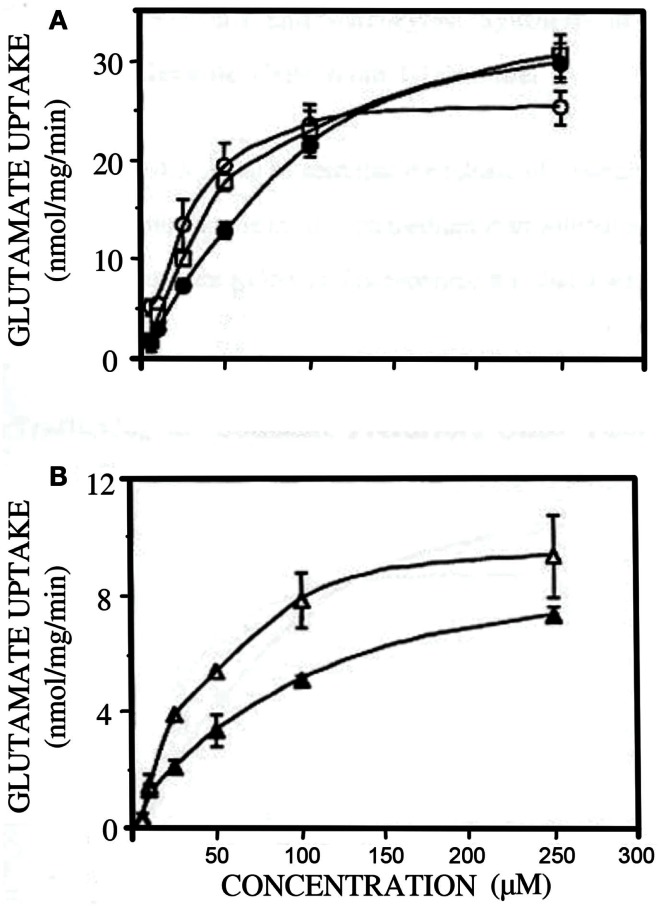
**Developmental changes in glutamate uptake in primary cultures of mouse astrocytes (A) and cerebral cortical neurons (B)**. **(A)** open circles, open squares and filled circles: 7, 18 and 30 days in culture; **(B)** open triangles 4 and closed triangles 8 days in culture. From Peng (1995).

The postnatal gliogenesis is accompanied and followed by many biochemical alterations. As could be expected, the activity of core astrocyte-specific enzymes depend upon the formation of astrocytes, although some enzymes that later become astrocyte-specific are neuronal during early development. The activity of the pyruvate carboxylase is very low in 8-day-old rat brain and only reaches adult activity after >30 days (Wilbur and Patel, 1974) (Figure 9). A fivefold higher pyruvate carboxylase activity in adult mouse brain than in newborn mouse brain was confirmed by Yu et al. (1983), and Yu (1984), showed a 10- to 15-fold increase in the activity of this enzyme in astrocyte *cultures* between the ages of 1 and 3 weeks. This contrasts a much faster development of glutamate uptake in cultured astrocytes, which is quite pronounced, although not mature, in 1-week-old cultures (Figure 10A). The activity of glutamine synthetase increases steeply during all the first 3 weeks of development both in *cultured* astrocytes and in brain *in vivo* (Hertz et al., 1978c; Patel et al., 1982). Glycogen content as well as activity of its degrading enzyme, glycogen phosphorylase, are low in brain at birth and increase during early postnatal development (Ferris and Himwich, 1946; Folbergrová, 1995).

The activities of many enzymes that are not specific for astrocytes also show drastic increases in activity during this period. This applies to glucose-metabolizing enzymes (e.g., hexokinase, aldolase, lactate dehydrogenase, phosphofructokinase, and PDH), which increase considerably in activity between the ages of ∼15 and ∼30 days (Takagaki, 1974; Wilbur and Patel, 1974; Land et al., 1977; Leong and Clark, 1984; – see also Figure 9). Synaptic mitochondria mature earlier (Almeida et al., 1995) than non-synaptic mitochondria (Bates et al., 1994), and cytosolic malate dehydrogenase (MDHc), which operates in the MAS but not in the TCA cycle, matures much more slowly than mitochondrial malate dehydrogenase (MDHm), which functions both in the MAS (Figure 1) and in the TCA cycle (Malik et al., 1993). Glutamate-metabolizing enzymes also show changes during the early postnatal period. Thus, the glutamate dehydrogenase activity falls between the ages of 2 and 3 weeks in astrocyte cultures, whereas that of aspartate aminotransferase increases (Hertz et al., 1978c). These observations point toward a slow postnatal development of the glutamine–glutamate (GABA) cycle, and perhaps decreased importance of GDH.

A three fourfold increase in cycle flux has been shown by ^13^C-NMR in rat brain cortex by Chowdhury et al. (2007a) between postnatal days 10 and 30, and energy metabolism in the glutamatergic and GABAergic neurons increased proportionately with cycle flux, leading to an ∼threefold increase in TCA cycle activity between postnatal days 10 and 30. Since, as was illustrated in Figure 8B, the amount of respiring brain tissue also increases hugely, *total* rate of energy metabolism in the rat brain cortex must increase about 10 times within these 20 days. That a small amount of astrocytic activity did occur, even at day 10 is indicated by the finding of detectable, although low incorporation of label from the astrocyte-specific substrate acetate into glutamate and GABA. Thus, postnatal day 10 must be close to the beginning of neuronal–astrocytic interactions involved in the glutamine–glutamate (GABA) cycle. Re-analysis of the acetate data may allow determination of the developmental pattern also of astrocytic TCA cycle flux (D. L. Rothman and K. L. Behar, personal communication). Measuring *rapid* incorporation of ^14^C from glucose into amino acids in rat brain, which also is an indication of glutamine–glutamate (GABA) cycle function, at many different developmental stages, Gaitonde and Richter (1966) and Patel and Balázs (1970) had also found a sharp increase between postnatal days 10 and 20, and that a maximum was not reached until around postnatal day 25. Maximum metabolic compartmentation between glutamate and glutamine, another indicator of glutamine–glutamate cycle activity was not found until a few days later. Thus old-style biochemical studies and cutting-edge ^13^C-NMR determinations have similarly identified the time period during which the glutamine–glutamate (GABA) cycle develops in the rat to between postnatal days 10 and 30.

Other aspects of TCA cycle function in brain are completed around postnatal day 15, indicated by maximum oxidative response in rat brain slices to stimuli at this age (Holtzman et al., 1982), and by sensory-evoked increases in brain glucose utilization *in vivo* by day 10 in barrel cortex (Melzer et al., 1994) and between days ∼13 to ∼18 in auditory and visual areas (Nehlig and de Vasconcelos, 1993). A functioning brain cortex is obviously working at that time, which is consistent with the completion of neurogenesis except at a few specific locations (Altman, 1969a). This is also exemplified by active GABAergic signaling at early neonatal stages (Lauder et al., 1986), and glutamatergic activation of synchronized spike waves in 3–5-day-old rats (Seki et al., 2012). Astrocytes are among the targets of glutamatergic signaling, which plays a role during astrocytic differentiation (Oppelt et al., 2004; Stipursky et al., 2012; Sun et al., 2013). Such an ontogenetic development from a purely neuronal nervous system to a neuronal–glial system also occurs during phylogenesis (Reichenbach and Pannicke, 2008). It is generally accepted that mammalian brain function should be studied in mammals, but far too often tissues from rats or mice younger than 4 weeks are studied.

In the absence of pyruvate carboxylase activity during early postnatal development (Figure 9) glutamate synthesis within the brain must be replaced by import of glutamate or a precursor. A relatively fast uptake of glutamine, glutamate, and GABA from the systemic circulation occurs across the blood-brain barrier at this age (Pardridge and Mietus, 1982; Al-Sarraf et al., 1997; Al-Sarraf, 2002), and it might be the source of neuronal amino acid transmitters. Also, during early postnatal development GLT1 is expressed in neurons (Shibata et al., 1996), although it later becomes astrocyte-specific. Glutamate oxidation by neurons occurs at much higher rates during early development, when little if any glutamate can be metabolized by astrocytes. This is illustrated in Figure 10B, showing a 50% reduction in rate of glutamate uptake in the glutamatergic cerebellar granule neurons between the ages of 4 and 8 days in culture (Peng, 1995). Similarly, other accounts of neuronal ability to oxidize glutamate may reflect the young age of the neurons (Olstad et al., 2007).

**Figure 11:**
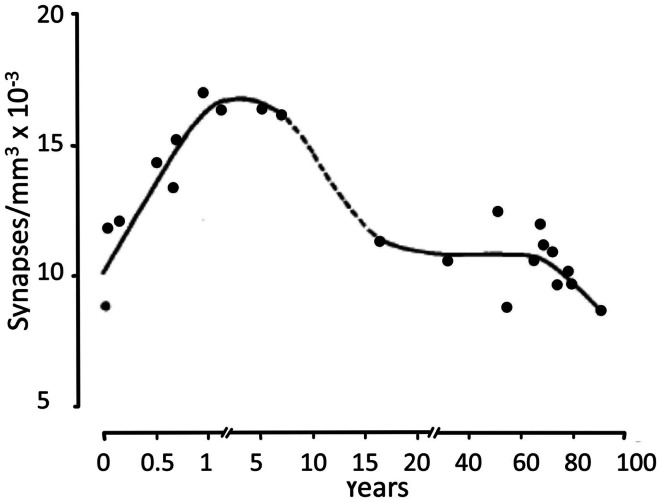
**Developmental changes in synaptic density in frontal gyrus, peaking at 12–15 months of age, and before or during adolescence decreasing to similar levels as in the newborn**. From Huttenlocher (1979).

The large increase in metabolic demand during the first postnatal month may partly reflect that instead of purely neuronal uptake of glutamate and GABA during very early postnatal development, virtually all glutamate and some GABA now becomes accumulated twice. The first uptake is of glutamate and GABA into astrocytes, where a large fraction is converted to glutamine in an energy-requiring process, and the second uptake is that of glutamine into neurons. However, this alone cannot explain a 10-fold increase in energy demand, and many other processes may also become very costly in energy. Thus, Na^+^,K^+^-ATPase-mediated uptake of K^+^ released during neuronal excitation into astrocytes seems also to precede neuronal re-accumulation of K^+^ in *adult astrocytes and brain* (Xu et al., 2013). Since the astrocytic K^+^ uptake also depends on glycogenolysis-activated signaling (to facilitate Na^+^ access to the Na^+^,K^+^-ATPase’s Na^+^-sensitive site), it must also be absent in neonatal brain. Greatly increased growth and branching of *neuronal* processes also occur during the first year (Conel, cited by Altman, 1967), and synaptic density increases, in frontal gyrus peaking at 12–15 months of age (Figure 11) and before or during adolescence decreasing to similar levels as in the newborn (Huttenlocher, 1979; Huttenlocher and Dabholkar, 1997).

Since the early postnatal rat brain functions well on the much smaller budget and without astrocytic participation in glutamate and GABA biosynthesis, one may ask which advantages could be associated with the developing dependence on astrocytic functions. One potential answer may be enhanced precision and more autonomy from the periphery. The changes may affect mental function, including learning. The day-old chick is a precocious animal. Its brain contains glycogen, and both glycogenolysis and glutamate formation, events occurring during glutamate production in the combined neuronal–astrocytic system, are essential for learning of a one-trial aversive memory task (Gibbs et al., 2007). Moreover, learning is inhibited by a glial-specific metabolic inhibitor (Ng et al., 1992). In this task the bird learns to associate a specific color on an artificial bead tainted with an aversively tasting compound and as a result later refuses to peck at beads of this color, even when untainted. Noradrenaline, released from locus coeruleus, acts mainly on astrocytes during learning in the day-old chick (Gibbs et al., 2008), but in the non-precocious newborn rat pups odor learning occurs via a direct noradrenaline effect on the neuronal mitral cells of the olfactory glomerulus (Wilson and Sullivan, 1991). In odor learning, pairing with an aversive stimulus has no negative effect until postnatal day 10–12 (Haroutunian and Campbell, 1979; Camp and Rudy, 1988; Raineki et al., 2010). This coincides with incipient glutamine–glutamate (GABA) cycle function in the brain. It would be interesting to know if disruption of glutamate production, and thus of glutamine supply to neurons, by the inhibitor of glycogenolysis DAB would prevent the effect of pairing with an aversive stimulus, but not odor learning as such. Moreover, in the chick learning task, memory formation can also be inhibited by pharmacological disruption of astrocytic gap junction permeability, and trafficking of glutamine through the astrocytic syncytium might play a role in connecting the visual stimulus with the aversive gustatory signal. Would an inhibitor of astrocytic gap junction affect odor learning as such and odor learning paired with an aversive stimulus differently in 12-day-old rats?

**Figure 12:**
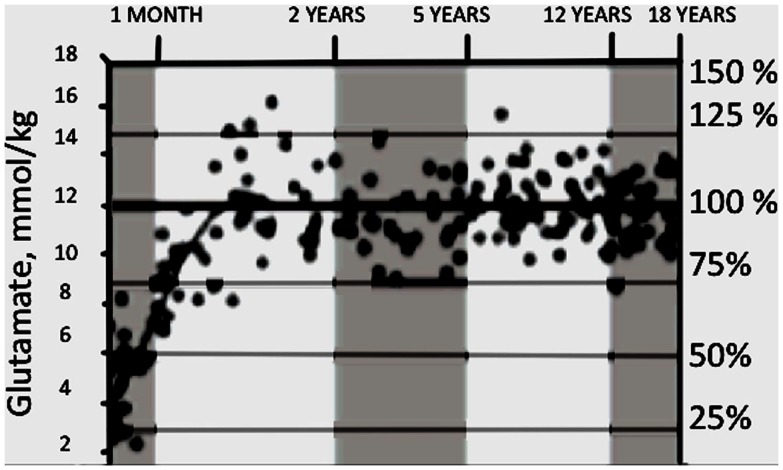
**Developmental changes in glutamate content in parietal/occipital gray matter measured by MRI in young children**. On percentage scale 100% refers to ages between 2 and 18 years. From Blüml et al. (2013).

In *human* brain cortex neurogenesis is also completed at birth in most brain regions (Bhardwaj et al., 2006), but as in many other animals, cortical gliogenesis occurs peri- and postnatally (Marn-Padilla, 2011). Recently glutamate content of different brain areas was studied in human children of different ages by magnetic resonance spectroscopy (MRS) in several brain regions (Blüml et al., 2013). Consistent with the late gliogenesis, the content of glutamate quadrupled between birth and 2 years of age (Figure 12) and subsequently remained stable at the adult value of about 12 mmol/kg. About one half of the change occurred during the first 3 months of life and most of the rest between 3 and 12 months of age.

Human learning *may* also provide a hint of functional gains that are time-wise, and *perhaps* causally, related to the change from a purely neuronal cerebral cortex to a cerebral cortex with the costly neuronal–astrocytic interaction, such as the glutamine–glutamate (GABA) cycle. Although disagreeing about mechanisms involved, both Rovee-Collier and Giles (2010) and Bauer and Nelson (Bauer et al., 2003; Bauer, 2006) similarly describe how early-maturing memory functions support gradual and exuberant learning of perceptual and motor skills, but memories are fragile and short-lived, as exemplified by our inability to recall events from early life. Only later-maturing modification(s) can support long-lasting representations of contextually specific events, relationships, temporal orders, and associations. Bauer (2006, 2008) as well as Nelson (1995) consider these differences as due to the operation of two different memory systems, implicit and explicit, with implicit memory being the unconscious memory function involved in motor skills. The development of explicit memory may occur via a pre-explicit system, and Nelson (1995) suggested that the development of explicit memory from implicit and pre-explicit memory may be associated with recruitment of additional specific brain structures. The simultaneous development and maturation of astrocytic functions and glutamine–glutamate (GABA) cycle operation together with the importance of astrocytic metabolic processes for aversive learning in the chick brain might suggest that the newly established, ubiquitous metabolic interactions between neurons and astrocytes could also play an important role in human learning.

## Concluding Remarks

The importance of the neuronal–astrocytic glutamine–glutamate (GABA) cycle in cerebral cortex is shown by the magnitude of this flux, equaling total rate of neuronal glucose oxidation. Although 85% of the cycle “only” serves to return previously released neurotransmitter glutamate and GABA, astrocytes contribute actively by accumulating the transmitters and converting them to glutamine, which can travel through gap junction-coupled astrocytes before its release. The astrocytic participation in the remaining 15% of the flux is even greater. They maintain an equilibrium between astrocytic biosynthesis and oxidative degradation, which perhaps also is astrocytic and is capable of establishing net synthesis or net degradation according to glutamate needs. The requirement of aspartate for maximum glutamate synthesis indicates that glutamate synthesis from α-ketoglutarate probably occurs by transamination, and pathway models for interaction between biosynthesis and oxidative degradation of both glutamate and GABA suggest that transamination may also be the default reaction for initiation of glutamate oxidation. Whether or not glutamate oxidation mainly occurs in astrocytes is perhaps the most important question, both for the viability of the suggested models (Figures 3B and 7) and for the functional importance of the glutamine–glutamate (GABA) cycle. Although the late development and maturation of the cycle has been realized for more than 40 years, the full consequences of this late and profound alteration in brain metabolism remain to be fully understood. They represent a development from purely neuronal processes to integrated neuronal and astrocytic activities, which may be functionally extremely important in brain function, including human mental activities. In practical terms it also means that mature brain function should not be investigated until full maturation has occurred, which in mice and rats is at a postnatal age of 3–4 weeks.

## Conflict of Interest Statement

The authors declare that the research was conducted in the absence of any commercial or financial relationships that could be construed as a potential conflict of interest.

## References

[B1] Abadom P. N. Scholefield P. G. (1962). Amino acid transport in brain cortex slices. I. The relation between energy production and the glucose-dependent transport of glycine. Can. J. Biochem. Physiol. 40, 1575–1590 10.1139/o62-178 14010520

[B2] Almeida A. Brooks K. J. Sammut I. Keelan J. Davey G. P. Clark J. B. (1995). Postnatal development of the complexes of the electron transport chain in synaptic mitochondria from rat brain. Dev. Neurosci. 17, 212–218 10.1159/000111289 8575340

[B3] Al-Sarraf H. (2002). Transport of ^14^C-gamma-aminobutyric acid into brain, cerebrospinal fluid and choroid plexus in neonatal and adult rats. Brain Res. Dev. Brain Res. 139, 121–129 10.1016/S0165-3806(02)00537-0 12480126

[B4] Al-Sarraf H. Preston J. E. Segal M. E. (1997). Changes in the kinetics of the acidic amino acid brain and CSF uptake during development in the rat. Brain Res. Dev. Brain Res. 102, 137–144 10.1016/S0165-3806(97)00075-8 9298241

[B5] Altman J. (1967). “Prenatal growth and differentiation of the mammalian brain, with implications for a morphological theory of memory,” in The Neurosciences – A Study Program, eds Quarton G. C. Melnechuk T. Schmitt F. O. (New York: The Rockefeller University Press), 723–743

[B6] Altman J. (1969a). Autoradiographic and histological studies of postnatal neurogenesis. IV. Cell proliferation and migration in the anterior forebrain, with special reference to persisting neurogenesis in the olfactory bulb. J. Comp. Neurol. 137, 433–457 10.1002/cne.901370404 5361244

[B7] Altman J. (1969b). “DNA metabolism and cell proliferation,” in Handbook of Neurochemistry, Vol. 2, ed. Lajtha A. (New York: Plenum Press), 137–182

[B8] Bak L. K. Zieminska E. Waagepetersen H. S. Schousboe A. Albrecht J. (2008). Metabolism of [U-^13^C]glutamine and [U-^13^C]glutamate in isolated rat brain mitochondria suggests functional phosphate-activated glutaminase activity in matrix. Neurochem. Res. 33, 273–278 10.1007/s11064-007-9471-1 17763943

[B9] Balazs R. (1965). Control of glutamate oxidation in brain and liver mitochondrial systems. Biochem. J. 95, 497–508 14340100 10.1042/bj0950497 PMC1214349

[B10] Bates T. E. Heales S. J. Davies S. E. Boakye P. Clark J. B. (1994). Effects of 1-methyl-4-phenylpyridinium on isolated rat brain mitochondria: evidence for a primary involvement of energy depletion. J. Neurochem. 63, 640–648 10.1046/j.1471-4159.1994.63020640.x 8035188

[B11] Bauer P. J. (2006). Constructing a past in infancy: a neuro-developmental account. Trends Cogn. Sci. (Regul. Ed.) 10, 175–181 10.1016/j.tics.2006.02.009 16537115

[B12] Bauer P. J. (2008). Toward a neuro-developmental account of the development of declarative memory. Dev. Psychobiol. 50, 19–31 10.1002/dev.20265 18085555

[B13] Bauer D. E. Jackson J. G. Genda E. N. Montoya M. M. Yudkoff M. Robinson M. B. (2012). The glutamate transporter, GLAST, participates in a macromolecular complex that supports glutamate metabolism. Neurochem. Int. 61, 566–574 10.1016/j.neuint.2012.01.013 22306776 PMC3350823

[B14] Bauer P. J. Wiebe S. A. Carver L. J. Waters J. M. Nelson C. A. (2003). Developments in long-term explicit memory late in the first year of life: behavioral and electrophysiological indices. Psychol. Sci. 14, 629–635 10.1046/j.0956-7976.2003.psci_1476.x 14629697

[B15] Benjamin A. M. (1987). Influence of Na^+^, K^+^, and Ca^2+^ on glutamine synthesis and distribution in rat brain cortex slices: a possible linkage of glutamine synthetase with cerebral transport processes and energetics in the astrocytes. J. Neurochem. 48, 115711–115764 10.1111/j.1471-4159.1987.tb05641.x 2434618

[B16] Benjamin A. M. Quastel J. H. (1972). Locations of amino acids in brain slices from the rat. Tetrodotoxin-sensitive release of amino acids. Biochem. J. 128, 631–646 4634833 10.1042/bj1280631 PMC1173815

[B17] Berkich D. A. Ola M. S. Cole J. Sweatt A. J. Hutson S. M. LaNoue K. F. (2007). Mitochondrial transport proteins of the brain. J. Neurosci. Res. 85, 3367–3377 10.1002/jnr.21500 17847082

[B18] Bhardwaj R. D. Curtis M. A. Spalding K. L. Buchholz B. A. Fink D. Björk-Eriksson T. (2006). Neocortical neurogenesis in humans is restricted to development. Proc. Natl. Acad. Sci. U.S.A. 103, 12564–12568 10.1073/pnas.0605177103 16901981 PMC1567918

[B19] Blot A. Billups D. Bjørkmo M. Quazi A. Z. Uwechue N. M. Chaudhry F. A. (2009). Functional expression of two system A glutamine transporter isoforms in rat auditory brainstem neurons. Neuroscience 164, 998–1008 10.1016/j.neuroscience.2009.09.015 19751803 PMC2789247

[B20] Blüml S. Wisnowski J. L. Nelson M. D. Jr. Paquette L. Gilles F. H. Kinney H. C. (2013). Metabolic maturation of the human brain from birth through adolescence: insights from in vivo magnetic resonance spectroscopy. Cereb. Cortex. [Epub ahead of print]. 10.1093/cercor/bhs283 PMC3888212 22952278

[B21] Boulland J. L. Osen K. K. Levy L. M. Danbolt N. C. Edwards R. H. Storm-Mathisen J. (2002). Cell-specific expression of the glutamine transporter SN1 suggests differences in dependence on the glutamine cycle. Eur. J. Neurosci. 15, 1615–1631 10.1046/j.1460-9568.2002.01995.x 12059969

[B22] Bröer A. Albers A. Setiawan I. Edwards R. H. Chaudhry F. A. Lang F. (2002). Regulation of the glutamine transporter SN1 by extracellular pH and intracellular sodium ions. J. Physiol. (Lond.) 539, 3–14 10.1113/jphysiol.2001.013303 11850497 PMC2290136

[B23] Camp L. L. Rudy J. W. (1988). Changes in the categorization of appetitive and aversive events during postnatal development of the rat. Dev. Psychobiol. 21, 25–42 10.1002/dev.420210103 3338626

[B24] Chamma I. Chevy Q. Poncer J. C. Lévi S. (2012). Role of the neuronal K-Cl co-transporter KCC2 in inhibitory and excitatory neurotransmission. Front. Cell. Neurosci. 6:5 10.3389/fncel.2012.00005 22363264 PMC3282916

[B25] Chhina N. Kuestermann E. Halliday J. Simpson L. J. MacDonald I. A. Bachelard H. S. (2001). Measurement of human tricarboxylic acid cycle rates during visual activation by ^13^C magnetic resonance spectroscopy. J. Neurosci. Res. 66, 737–746 10.1002/jnr.10053 11746397

[B26] Chowdhury G. M. Gupta M. Gibson K. M. Patel A. B. (2007a). Altered cerebral glucose and acetate metabolism in succinic semialdehyde dehydrogenase-deficient mice: evidence for glial dysfunction and reduced glutamate/glutamine cycling. J. Neurochem. 103, 2077–2091 10.1111/j.1471-4159.2007.04887.x 17854388

[B27] Chowdhury G. M. Patel A. B. Mason G. F. Rothman D. L. Behar K. L. (2007b). Glutamatergic and GABAergic neurotransmitter cycling and energy metabolism in rat cerebral cortex during postnatal development. J. Cereb. Blood Flow Metab. 27, 1895–1907 10.1038/sj.jcbfm.9600490 17440492

[B28] Cruz N. F. Ball K. K. Dienel G. A. (2007). Functional imaging of focal brain activation in conscious rats: impact of [^14^C]glucose metabolite spreading and release. J. Neurosci. Res. 85, 3254–3266 10.1002/jnr.21436 17265468

[B29] Curtis D. R. Phillis J. W. Watkins J. C. (1960). The chemical excitation of spinal neurones by certain acidic amino acids. J. Physiol. (Lond.) 150, 656–682 13813400 10.1113/jphysiol.1960.sp006410 PMC1363189

[B30] Danbolt N. C. (2001). Glutamate uptake. Prog. Neurobiol. 65, 1–105 10.1016/S0301-0082(00)00067-8 11369436

[B31] Derouiche A. (2004). “The perisynaptic astrocyte process as a glial compartment-immunolabelling for glutamine synthetase and other glial markers,” in Non-Neuronal Cells of the Nervous System, Function and Dysfunction, Vol. 1, ed. Hertz L. (Amsterdam: Elsevier), 147–163

[B32] Dickens F. Greville G. D. (1935). The metabolism of normal and tumour tissue: neutral salt effects. Biochem. J. 29, 1468–1483 16745812 10.1042/bj0291468 PMC1266648

[B33] Dienel G. A. Ball K. K. Cruz N. F. (2007). A glycogen phosphorylase inhibitor selectively enhances local rates of glucose utilization in brain during sensory stimulation of conscious rats: implications for glycogen turnover. J. Neurochem. 102, 466–478 10.1111/j.1471-4159.2007.04595.x 17442042 PMC2822402

[B34] Dienel G. A. Wang R. Y. Cruz N. F. (2002). Generalized sensory stimulation of conscious rats increases labeling of oxidative pathways of glucose metabolism when the brain glucose-oxygen uptake ratio rise. J. Cereb. Blood Flow Metab. 22, 1490–1502 10.1097/00004647-200212000-00009 12468893

[B35] Eid T. Behar K. Dhaher R. Bumanglag A. V. Lee T. S. (2012). Roles of glutamine synthetase inhibition in epilepsy. Neurochem. Res. 37, 2339–2350 10.1007/s11064-012-0766-5 22488332 PMC3731630

[B36] Eriksson G. Peterson A. Iverfeldt K. Walum E. (1995). Sodium-dependent glutamate uptake as an activator of oxidative metabolism in primary astrocyte cultures from newborn rat. Glia 15, 152–156 10.1002/glia.440150207 8567066

[B37] Exposito I. Sanz B. Porras A. Mora F. (1994). Effects of apomorphine and L-methionine sulphoximine on the release of excitatory amino acid neurotransmitters and glutamine in the striatum of the conscious rat. Eur. J. Neurosci. 6, 287–291 10.1111/j.1460-9568.1994.tb00271.x 7909484

[B38] Ferris S. Himwich H. E. (1946). The effect of hypoglycemia and age on the glycogen content of the various parts of the feline central nervous system. Am. J. Physiol. 146, 389–393 20989248 10.1152/ajplegacy.1946.146.3.389

[B39] Florey E. (1956). An inhibitory and an excitatory factor of mammalian central nervous system and their action on a single sensory neuron. Arch. Int. Physiol. 62, 33–53 10.3109/13813455409145367 13149232

[B40] Folbergrová J. (1995). Glycogen phosphorylase activity in the cerebral cortex of rats during development: effect of homocysteine-induced seizures. Brain Res. 694, 128–132 10.1016/0006-8993(95)00805-Z 8974635

[B41] Frigerio F. Karaca M. De Roo M. Mlynárik V. Skytt D. M. Carobbio S. (2012). Deletion of glutamate dehydrogenase 1 (Glud1) in the central nervous system affects glutamate handling without altering synaptic transmission. J. Neurochem. 123, 342–348 10.1111/j.1471-4159.2012.07933.x 22924626

[B42] Gaitonde M. K. Richter D. (1966). Changes with age in the utilization of glucose carbon in liver and brain. J. Neurochem. 13, 1309–1316 10.1111/j.1471-4159.1966.tb04293.x 5962013

[B43] Garrison J. C. Borland M. K. (1979). Regulation of mitochondrial pyruvate carboxylation and gluconeogenesis in rat hepatocytes via an alpha-adrenergic, adenosine 3′:5′-monophosphate-independent mechanism. J. Biol. Chem. 254, 1129–1133 33183

[B44] Ge W. P. Miyawaki A. Gage F. H. Jan L. Y. (2012). Local generation of glia is a major astrocyte source in postnatal cortex. Nature 484, 376–380 10.1038/nature10959 22456708 PMC3777276

[B45] Ghosh J. J. Quastel J. H. (1954). Narcotics and brain respiration. Nature 174, 28–31 10.1038/174028a0 13176425

[B46] Gibbs M. E. Hutchinson D. Hertz L. (2008). Astrocytic involvement in learning and memory consolidation. Neurosci. Biobehav. Rev. 32, 927–944 10.1016/j.neubiorev.2008.02.001 18462796

[B47] Gibbs M. E. Lloyd H. G. Santa T. Hertz L. (2007). Glycogen is a preferred glutamate precursor during learning in 1-day-old chick: biochemical and behavioral evidence. J. Neurosci. Res. 85, 3326–3333 10.1002/jnr.21307 17455305

[B48] Gruetter R. Seaquist E. R. Ugurbil K. (2001). A mathematical model of compartmentalized neurotransmitter metabolism in the human brain. Am. J. Physiol. Endocrinol. Metab. 281, E100–E112 11404227 10.1152/ajpendo.2001.281.1.E100

[B49] Haroutunian V. Campbell B. A. (1979). Emergence of interoceptive and exteroceptive control of behavior in rats. Science 205, 927–929 10.1126/science.472715 472715

[B50] Hertz L. (2011a). Brain glutamine synthesis requires neuronal aspartate: a commentary. J. Cereb. Blood Flow Metab. 231, 384–387 10.1038/jcbfm.2010.199 21063427 PMC3049474

[B51] Hertz L. (2011b). Astrocytic energy metabolism and glutamate formation – relevance for ^13^C-NMR spectroscopy and importance of cytosolic/mitochondrial trafficking. Magn. Reson. Imaging 29, 1319–1329 10.1016/j.mri.2011.04.013 21820830

[B52] Hertz L. Bock E. Schousboe A. (1978c). GFA content, glutamate uptake and activity of glutamate metabolizing enzymes in differentiating mouse astrocytes in primary cultures. Dev. Neurosci. 1, 226–238 10.1159/000112577

[B53] Hertz L. Drejer J. Schousboe A. (1988). Energy metabolism in glutamatergic neurons, GABAergic neurons and astrocytes in primary cultures. Neurochem. Res. 13, 605–610 10.1007/BF00973275 2901049

[B54] Hertz L. Hertz E. (2003). Cataplerotic TCA cycle flux determined as glutamate-sustained oxygen consumption in primary cultures of astrocytes. Neurochem. Int. 43, 355–361 10.1016/S0197-0186(03)00022-6 12742079

[B55] Hertz L. O’Dowd B. S. Ng K. T. Gibbs M. E. (2003). Reciprocal changes in forebrain contents of glycogen and of glutamate/glutamine during early memory consolidation in the day-old chick. Brain Res. 994, 226–233 10.1016/j.brainres.2003.09.044 14642648

[B56] Hertz L. Peng L. Dienel G. A. (2007). Energy metabolism in astrocytes: high rate of oxidative metabolism and spatiotemporal dependence on glycolysis/glycogenolysis. Cereb. Blood Flow Metab. 27, 219–249 10.1038/sj.jcbfm.9600343 16835632

[B57] Hertz L. Schousboe A. Boechler N. Mukerji S. Fedoroff S. (1978b). Kinetic characteristics of the glutamate uptake into normal astrocytes in cultures. Neurochem. Res. 3, 1–14 10.1007/BF00965577 683409

[B58] Hertz L. Wu P. H. Schousboe A. (1978a). Evidence for net uptake of GABA into mouse astrocytes in primary cultures – its sodium dependence and potassium independence. Neurochem. Res. 3, 313–323 10.1007/BF00965577 745651

[B59] Hertz L. Xu J. Song D. Du T. Yan E. Peng L. (2013). Brain glycogenolysis, adrenoceptors, pyruvate carboxylase, Na^+^,K^+^-ATPase and Marie E. Gibbs’ pioneering learning studies. Front. Integr. Neurosci. 7:20 10.3389/fnint.2013.00020 23565080 PMC3615183

[B60] Hof P. R. Pascale E. Magistretti P. J. (1988). K^+^ at concentrations reached in the extracellular space during neuronal activity promotes a Ca^2+^-dependent glycogen hydrolysis in mouse cerebral cortex. J. Neurosci. 8, 1922–1928 3385482 10.1523/JNEUROSCI.08-06-01922.1988 PMC6569342

[B61] Holtzman D. Olson J. Zamvil S. Nguyen H. (1982). Maturation of potassium-stimulated respiration in rat cerebral cortical slices. J. Neurochem. 39, 274–276 10.1111/j.1471-4159.1982.tb04734.x 7086416

[B62] Hutson S. M. Berkich D. Drown P. Xu B. Aschner M. LaNoue K. F. (1998). Role of branched-chain aminotransferase isoenzymes and gabapentin in neurotransmitter metabolism. J. Neurochem. 71, 863–874 10.1046/j.1471-4159.1998.71020863.x 9681479

[B63] Hutson S. M. Cole J. T. Sweatt A. J. LaNoue K. F. (2008). Is the anaplerotic enzyme pyruvate carboxylase (PC) only expressed in astrocytes? J. Neurochem. 104, S58

[B64] Huttenlocher P. R. (1979). Synaptic density in human frontal cortex – developmental changes and effects of aging. Brain Res. 163, 195–205 10.1016/0006-8993(79)90349-4 427544

[B65] Huttenlocher P. R. Dabholkar A. S. (1997). Regional differences in synaptogenesis in human cerebral cortex. J. Comp. Neurol. 387, 167–178 10.1002/(SICI)1096-9861(19971020)387:2<167::AID-CNE1>3.0.CO;2-Z 9336221

[B66] Hyder F. Fulbright R. K. Shulman R. G. Rothman D. L. (2013). Glutamatergic function in the resting awake human brain is supported by uniformly high oxidative energy. J. Cereb. Blood Flow Metab. 33, 339–347 10.1038/jcbfm.2012.207 23299240 PMC3587823

[B67] Jenstad M. Quazi A. Z. Zilberter M. Haglerød C. Berghuis P. Saddique N. (2009). System A transporter SAT2 mediates replenishment of dendritic glutamate pools controlling retrograde signaling by glutamate. Cereb. Cortex 19, 1092–1106 10.1093/cercor/bhn151 18832333

[B68] Kanamori K. Ross B. D. (2006). Kinetics of glial glutamine efflux and the mechanism of neuronal uptake studied in vivo in mildly hyperammonemic rat brain. J. Neurochem. 99, 1103–1113 10.1111/j.1471-4159.2006.04152.x 17081141

[B69] Kasischke K. A. Vishwasrao H. D. Fisher P. J. Zipfel W. R. Webb W. W. (2004). Neural activity triggers neuronal oxidative metabolism followed by astrocytic glycolysis. Science 305, 99–103 10.1126/science.1096485 15232110

[B70] Kaufman E. E. Driscoll B. F. (1993). Evidence for cooperativity between neurons and astroglia in the regulation of CO_2_ fixation in vitro. Dev. Neurosci. 15, 299–305 10.1159/000111348 7805582

[B71] Kurz G. M. Wiesinger H. Hamprecht B. (1993). Purification of cytosolic malic enzyme from bovine brain, generation of monoclonal antibodies, and immunocytochemical localization of the enzyme in glial cells of neural primary cultures. J. Neurochem. 60, 1467–1474 10.1111/j.1471-4159.1993.tb03309.x 8455034

[B72] Lai J. C. Clark J. B. (1976). Preparation and properties of mitochondria derived from synaptosomes. Biochem. J. 154, 423–432 938457 10.1042/bj1540423 PMC1172723

[B73] Land J. M. Booth R. F. Berger R. Clark J. B. (1977). Development of mitochondrial energy metabolism in rat brain. Biochem. J. 164, 339–348 880241 10.1042/bj1640339 PMC1164798

[B74] LaNoue K. F. Carson V. Berkich D. A. Hutson S. (2007). “Mitochondrial/cytosolic interactions via metabolite shuttles and transporters,” in Handbook of Neurochemistry and Molecular Neurobiology, Vol. 2, eds Lajtha A. Gibson G. E. Dienel G. A. (Berlin: Springer Verlag), 5616–5689

[B75] Lauder J. M. Han V. K. Henderson P. Verdoorn T. Towle A. C. (1986). Prenatal ontogeny of the GABAergic system in the rat brain: an immunocytochemical study. Neuroscience 19, 465–493 10.1016/0306-4522(86)90275-7 3022187

[B76] Lebon V. Petersen K. F. Cline G. W. Shen J. Mason G. F. Dufour S. (2002). Astroglial contribution to brain energy metabolism in humans revealed by ^13^C nuclear magnetic resonance spectroscopy: elucidation of the dominant pathway for neurotransmitter glutamate repletion and measurement of astrocytic oxidative metabolism. J. Neurosci. 22, 1523–1531 11880482 10.1523/JNEUROSCI.22-05-01523.2002 PMC2995528

[B77] Leong S. F. Clark J. B. (1984). Regional enzyme development in rat brain. Enzymes associated with glucose utilization. Biochem. J. 218, 131–138 6712609 10.1042/bj2180131 PMC1153316

[B78] Li M. Li C. Allen A. Stanley C. A. Smith T. J. (2012a). The structure and allosteric regulation of mammalian glutamate dehydrogenase. Arch. Biochem. Biophys. 519, 69–80 10.1016/j.abb.2011.10.015 22079166 PMC3294041

[B79] Li B. Hertz L. Peng L. (2012b). Aralar mRNA and protein levels in neurons and astrocytes freshly isolated from young and adult mouse brain and in maturing cultured astrocytes. Neurochem. Int. 61, 325–332 10.1016/j.neuint.2012.07.018 23017600

[B80] Lin Y. Stephenson M. C. Xin L. Napolitano A. Morris P. G. (2012). Investigating the metabolic changes due to visual stimulation using functional proton magnetic resonance spectroscopy at 7 T. J. Cereb. Blood Flow Metab. 32, 1484–1495 10.1038/jcbfm.2012.33 22434070 PMC3421086

[B81] Lipsett M. N. Crescitelli F. (1950). The effects of increased potassium concentration on the metabolism of rat cerebral cortical slices. Arch. Biochem. 28, 329–337 14790774

[B82] Löscher W. Puskarjov M. Kaila K. (2013). Cation-chloride cotransporters NKCC1 and KCC2 as potential targets for novel antiepileptic and antiepileptogenic treatments. Neuropharmacology 69, 62–74 10.1016/j.neuropharm.2012.05.045 22705273

[B83] Lovatt D. Sonnewald U. Waagepetersen H. S. Schousboe A. He W. Lin J. H. (2007). The transcriptome and metabolic gene signature of protoplasmic astrocytes in the adult murine cortex. J. Neurosci. 27, 12255–12266 10.1523/JNEUROSCI.3404-07.2007 17989291 PMC6673251

[B84] Ma D. Lu P. Yan C. Fan C. Yin P. Wang J. (2012). Structure and mechanism of a glutamate-GABA antiporter. Nature 483, 632–636 10.1038/nature10917 22407317

[B85] Magistretti P. J. (1988). Regulation of glycogenolysis by neurotransmitters in the central nervous system. Diabetes Metab. 14, 237–246 2900788

[B86] Malik P. McKenna M. C. Tildon J. T. (1993). Regulation of malate dehydrogenases from neonatal, adolescent, and mature rat brain. Neurochem. Res. 18, 247–257 10.1007/BF00969080 8479597

[B87] Mangia S. Giove F. Dinuzzo M. (2012). Metabolic pathways and activity-dependent modulation of glutamate concentration in the human brain. Neurochem. Res. 37, 2554–2561 10.1007/s11064-012-0848-4 22846967 PMC3489977

[B88] Mangia S. Tkác I. Gruetter R. Van de Moortele P. F. Maraviglia B. Ugurbil K. (2007). Sustained neuronal activation raises oxidative metabolism to a new steady-state level: evidence from 1H NMR spectroscopy in the human visual cortex. J. Cereb. Blood Flow Metab. 27, 1055–1063 17033694 10.1038/sj.jcbfm.9600401

[B89] Marcaggi P. Coles J. A. (2000). A Cl^−^ cotransporter selective for ${\text{NH}}_{4}^{+}$ over K^+^ in glial cells of bee retina. J. Gen. Physiol. 116, 125–142 10.1085/jgp.116.2.125 10919861 PMC2229498

[B90] Marn-Padilla M. (2011). The Human Brain. Berlin: Springer

[B91] McKenna M. C. (2011). Glutamate dehydrogenase in brain mitochondria: do lipid modifications and transient metabolon formation influence enzyme activity? Neurochem. Int. 59, 525–533 10.1016/j.neuint.2011.07.003 21771624 PMC3459329

[B92] McKenna M. C. (2012). Substrate competition studies demonstrate oxidative metabolism of glucose, glutamate, glutamine, lactate and 3-hydroxybutyrate in cortical astrocytes from rat brain. Neurochem. Res. 37, 2613–2626 10.1007/s11064-012-0901-3 23079895 PMC3547390

[B93] McKenna M. C. Sonnewald U. Huang X. Stevenson J. Zielke H. R. (1996). Exogenous glutamate concentration regulates the metabolic fate of glutamate in astrocytes. J. Neurochem. 66, 386–393 10.1046/j.1471-4159.1996.66010386.x 8522979

[B94] McKenna M. C. Tildon J. T. Stevenson J. H. Huang X. Kingwell K. G. (1995). Regulation of mitochondrial and cytosolic malic enzymes from cultured rat brain astrocytes. Neurochem. Res. 20, 1491–1501 10.1007/BF00970599 8789613

[B95] McLennan H. (1976). The autoradiographic localization of L-[^3^H] glutamate in rat brain tissue. Brain Res. 115, 139–144 10.1016/0006-8993(76)90828-3 974737

[B96] Melzer P. Welker E. Dörfl J. Van der Loos H. (1994). Maturation of the neuronal metabolic response to vibrissa stimulation in the developing whisker-to-barrel pathway of the mouse. Brain Res. Dev. Brain Res. 77, 227–250 10.1016/0165-3806(94)90199-6 8174231

[B97] Michaeli S. Fait A. Lagor K. Nunes-Nesi A. Grillich N. Yellin A. (2011). A mitochondrial GABA permease connects the GABA shunt and the TCA cycle, and is essential for normal carbon metabolism. Plant J. 67, 485–498 10.1111/j.1365-313X.2011.04612.x 21501262

[B98] Mori K. Yamagami S. Kawakita Y. (1970). Thymidine metabolism and deoxyribonucleic acid synthesis in the developing rat brain. J. Neurochem. 17, 835–843 10.1111/j.1471-4159.1970.tb02237.x 5426674

[B99] Nagaraja T. N. Brookes N. (1998). Intracellular acidification induced by passive and active transport of ammonium ions in astrocytes. Am. J. Physiol. 274, C883–C891 9575784 10.1152/ajpcell.1998.274.4.C883

[B100] Nehlig A. de Vasconcelos A. (1993). Glucose and ketone body utilization by the brain of neonatal rats. Prog. Neurobiol. 40, 163–221 10.1016/0301-0082(93)90022-K 8430212

[B101] Nelson C. A. (1995). The nature of early memory. Prev. Med. 27, 172–179 10.1006/pmed.1998.0272 9578990

[B102] Ng K. T. Gibbs M. E. Gibbs C. L. Sedman G. Syková E. Svoboda J. (1992). Ion involvement in memory formation: the potential role of astrocytes. Prog. Brain Res. 94, 109–115 10.1016/S0079-6123(08)61743-4 1283788

[B103] Nicklas W. J. (1983). “Relative contributions of neurons and glia to metabolism of glutamate and GABA,” in Glutamine. Glutamate and GABA in the Central Nervous System, eds Hertz L. Kvamme E. McGeer E. Schousboe A. (New York: Alan R. Liss. Inc.), 219–231

[B104] Norenberg M. D. Martinez-Hernandez A. (1979). Fine structural localization of glutamine synthetase in astrocytes of rat brain. Brain Res. 161, 303–310 10.1016/0006-8993(79)90071-4 31966

[B105] Okamoto S. (1951). Epileptogenic action of glutamate directly applied into the brains of animals and inhibitory effects of protein and tissue emulsions on its action. J. Physiol. Soc. Jpn. 13, 555–562

[B106] Olstad E. Olsen G. M. Qu H. Sonnewald U. (2007). Pyruvate recycling in cultured neurons from cerebellum. J. Neurosci. Res. 85, 3318–3325 10.1002/jnr.21208 17304574

[B107] Ondo J. G. Pass K. A. (1976). The effects of neurally active amino acids on prolactin secretion. Endocrinology 98, 1248–1252 10.1210/endo-98-5-1248 4297

[B108] Ondo J. G. Pass K. A. Baldwin R. (1976). The effects of neurally active amino acids on pituitary gonadotropin secretion. Neuroendocrinology 21, 79–87 10.1159/000122514 1004688

[B109] Oppelt D. Rodnight R. Horn J. Fitarelli D. Kommers T. Oliveira D. (2004). Role of intracellular calcium stores on the effect of metabotropic glutamate receptors on phosphorylation of glial fibrillary acidic protein in hippocampal slices from immature rats. Neurochem. Res. 29, 1541–1545 10.1023/B:NERE.0000029567.68068.ab 15260132

[B110] Öz G. Berkich D. A. Henry P. G. Xu Y. LaNoue K. Hutson S. M. (2004). Neuroglial metabolism in the awake rat brain: CO2 fixation increases with brain activity. J. Neurosci. 24, 11273–11279 10.1523/JNEUROSCI.3564-04.2004 15601933 PMC6730363

[B111] Öz G. Tesfaye N. Kumar A. Deelchand D. K. Eberly L. E. Seaquist E. R. (2012). Brain glycogen content and metabolism in subjects with type 1 diabetes and hypoglycemia unawareness. J. Cereb. Blood Flow Metab. 32, 256–263 10.1038/jcbfm.2011.138 21971353 PMC3272603

[B112] Palaiologos G. Hertz L. Schousboe A. (1988). Evidence that aspartate aminotransferase activity and ketodicarboxylate carrier function are essential for biosynthesis of transmitter glutamate. J. Neurochem. 51, 317–320 10.1111/j.1471-4159.1988.tb04872.x 2898006

[B113] Pardo B. Rodrigues T. B. Contreras L. Garzón M. Llorente-Folch I. Kobayashi K. (2011). Brain glutamine synthesis requires neuronal-born aspartate as amino donor for glial glutamate formation. J. Cereb. Blood Flow Metab. 31, 90–101 10.1038/jcbfm.2010.146 20736955 PMC3049464

[B114] Pardridge W. M. Mietus L. J. (1982). Kinetics of neutral amino acid transport through the blood-brain barrier of the newborn rabbit. J. Neurochem. 38, 955–962 10.1111/j.1471-4159.1982.tb08663.x 7062043

[B115] Patel A. B. de Graaf R. A. Mason G. F. Kanamatsu T. Rothman D. L. Shulman R. G. (2004). Glutamatergic neurotransmission and neuronal glucose oxidation are coupled during intense neuronal activation. J. Cereb. Blood Flow Metab. 24, 972–985 10.1097/01.WCB.0000126234.16188.71 15356418

[B116] Patel A. B. de Graaf R. A. Mason G. F. Rothman D. L. Shulman R. G. Behar K. L. (2005). The contribution of GABA to glutamate/glutamine cycling and energy metabolism in the rat cortex in vivo. Proc. Natl. Acad. Sci. U.S.A. 102, 5588–5593 10.1073/pnas.0506351102 15809416 PMC556230

[B117] Patel A. J. Balázs R. (1970). Manifestation of metabolic compartmentation during the maturation of the rat brain. J. Neurochem. 17, 955–971 10.1111/j.1471-4159.1970.tb02249.x 5448598

[B118] Patel A. J. Hunt A. Gordon R. D. Balázs R. (1982). The activities in different neural cell types of certain enzymes associated with the metabolic compartmentation of glutamate. Brain Res. 256, 3–11 6124308 10.1016/0165-3806(82)90091-8

[B119] Peng L. (1995). Metabolic Trafficking Between Neurons and Astrocytes. Ph.D. thesis, University of Saskatchewan, Saskatoon, SK

[B120] Peng L. Swanson R. A. Hertz L. (2001). Effects of L-glutamate, D-aspartate, and monensin on glycolytic and oxidative glucose metabolism in mouse astrocyte cultures: further evidence that glutamate uptake is metabolically driven by oxidative metabolism. Neurochem. Int. 38, 437–443 10.1016/S0197-0186(00)00104-2 11222924

[B121] Perez E. L. Lauritzen F. Wang Y. Lee T. S. Kang D. Zaveri H. P. (2012). Evidence for astrocytes as a potential source of the glutamate excess in temporal lobe epilepsy. Neurobiol. Dis. 47, 331–337 10.1016/j.nbd.2012.05.010 22659305 PMC3392431

[B122] Petroff O. A. Errante L. D. Rothman D. L. Kim J. H. Spencer D. D. (2002a). Neuronal and glial metabolite content of the epileptogenic human hippocampus. Ann. Neurol. 52, 635–642 10.1002/ana.10360 12402262

[B123] Petroff O. A. Errante L. D. Rothman D. L. Kim J. H. Spencer D. D. (2002b). Glutamate-glutamine cycling in the epileptic human hippocampus. Epilepsia 43, 703–710 10.1046/j.1528-1157.2002.38901.x 12102672

[B124] Raineki C. Pickenhagen A. Roth T. L. Babstock D. M. McLean J. H. Harley C. W. (2010). The neurobiology of infant maternal odor learning. Braz. J. Med. Biol. Res. 43, 914–919 10.1590/S0100-879X2010007500090 20835686 PMC3602791

[B125] Ramos M. del Arco A. Pardo B. Martínez-Serrano A. Martínez-Morales J. R. Kobayashi K. (2003). Developmental changes in the Ca^2+^-regulated mitochondrial aspartate-glutamate carrier aralar1 in brain and prominent expression in the spinal cord. Brain Res. Dev. Brain Res. 143, 33–46 10.1016/S0165-3806(03)00097-X 12763579

[B126] Rao V. L. Murthy C. R. (1993). Uptake and metabolism of glutamate and aspartate by astroglial and neuronal preparations of rat cerebellum. Neurochem. Res. 18, 647–654 10.1007/BF00966777 8099717

[B127] Reichenbach A. Pannicke T. (2008). Neuroscience. A new glance at glia. Science 322, 693–694 10.1126/science.1166197 18974341

[B128] Richter K. Hamprecht B. Scheich H. (1996). Ultrastructural localization of glycogen phosphorylase predominantly in astrocytes of the gerbil brain. Glia 17, 263273 10.1002/(SICI)1098-1136(199608)17:4<263::AID-GLIA1>3.0.CO;2-0 8856323

[B129] Roberts E. (1956). “Formation and liberation of γ-aminobutyric acid in brain,” in Progress in Neurobiology. I. Neurochemistry, eds Korey S. R. Nurnberger J. I. (New York: Hoeber-Harper), 11–25 13359518

[B130] Rothman D. L. De Feyter H. M. de Graaf R. A. Mason G. F. Behar K. L. (2011). ^13^C MRS studies of neuroenergetics and neurotransmitter cycling in humans. NMR Biomed. 24, 943–957 10.1002/nbm.1772 21882281 PMC3651027

[B131] Rothman D. L. De Feyter H. M. Maciejewski P. K. Behar K. L. (2012). Is there in vivo evidence for amino acid shuttles carrying ammonia from neurons to astrocytes? Neurochem. Res. 37, 2597–2612 10.1007/s11064-012-0898-7 23104556 PMC3702378

[B132] Rovee-Collier C. Giles A. (2010). Why a neuromaturational model of memory fails: exuberant learning in early infancy. Behav. Processes 83, 197–206 10.1016/j.beproc.2009.11.013 19945516 PMC2823839

[B133] Schousboe A. (1972). Development of potassium effects on ion concentrations and indicator spaces in rat brain-cortex slices during postnatal ontogenesis. Exp. Brain Res. 15, 521–531 10.1007/BF00236406 4673705

[B134] Schousboe A. Lisy V. Hertz L. (1976). Postnatal alterations in effects of potassium on uptake and release of glutamate and GABA in rat brain cortex slices. J. Neurochem. 26, 1023–1027 10.1111/j.1471-4159.1976.tb06494.x 1271061

[B135] Schousboe A. Svenneby G. Hertz L. (1977). Uptake and metabolism of glutamate in astrocytes cultured from dissociated mouse brain hemispheres. J. Neurochem. 29, 999–1005 10.1111/j.1471-4159.1977.tb06503.x 23414

[B136] Seki M. Kobayashi C. Takahashi N. Matsuki N. Ikegaya Y. (2012). Synchronized spike waves in immature dentate gyrus networks. Eur. J. Neurosci. 35, 673–681 10.1111/j.1460-9568.2012.07995.x 22332872

[B137] Shank R. P. Bennett G. S. Freytag S. O. Campbel G. L. (1985). Pyruvate carboxylase: an astrocyte-specific enzyme implicated in the replenishment of amino acid neurotransmitter pools. Brain Res. 329, 364–367 10.1016/0006-8993(85)90552-9 3884090

[B138] Shibata T. Watanabe M. Tanaka K. Wada K. Inoue Y. (1996). Dynamic changes in expression of glutamate transporter mRNAs in developing brain. Neuroreport 7, 705–709 10.1097/00001756-199601310-00055 8733726

[B139] Sibson N. R. Dhankhar A. Mason G. F. Rothman D. L. Behar K. L. Shulman R. G. (1998). Stoichiometric coupling of brain glucose metabolism and glutamatergic neuronal activity. Proc. Natl. Acad. Sci. U.S.A. 95, 316–321 10.1073/pnas.95.1.316 9419373 PMC18211

[B140] Sickmann H. M. Walls A. B. Schousboe A. Bouman S. D. Waagepetersen H. S. (2009). Functional significance of brain glycogen in sustaining glutamatergic neurotransmission. J. Neurochem. 109(Suppl. 1), 80–86 10.1111/j.1471-4159.2009.05915.x 19393012

[B141] Song D. Du T. Li B. Cai L. Gu L. Li H. (2008). Astrocytic alkalinization by therapeutically relevant lithium concentrations: implications for myo-inositol depletion. Psychopharmacology (Berl.) 200, 187–195 10.1007/s00213-008-1194-8 18506424

[B142] Song D. Li B. Yan E. Man Y. Wolfson M. Chen Y. (2012). Chronic treatment with anti-bipolar drugs causes intracellular alkalinization in astrocytes, altering their functions. Neurochem. Res. 37, 2524–2540 10.1007/s11064-012-0837-7 22965852

[B143] Stipursky J. Spohr T. C. Sousa V. O. Gomes F. C. (2012). Neuron-astroglial interactions in cell-fate commitment and maturation in the central nervous system. Neurochem. Res. 37, 2402–2418 10.1007/s11064-012-0798-x 22614925

[B144] Storm-Mathisen J. Danbolt N. C. Rothe F. Torp R. Zhang N. Aas J. E. (1992). Ultrastructural immunocytochemical observations on the localization, metabolism and transport of glutamate in normal and ischemic brain tissue. Prog. Brain Res. 94, 225–241 10.1016/S0079-6123(08)61753-7 1363142

[B145] Storm-Mathisen J. Ottersen O. P. (1983). “Immunohistochemistry of glutamate and GABA,” in Glutamine. Glutamate and GABA in the Central Nervous System, eds Hertz L. Kvamme E. McGeer E. Schousboe A. (New York: Alan R. Liss. Inc.), 185–201

[B146] Subbarao K. V. Hertz L. (1990). Effect of adrenergic agonists on glycogenolysis in primary cultures of astrocytes. Brain Res. 536, 220–226 10.1016/0006-8993(90)90028-A 2085749

[B147] Sun W. McConnell E. Pare J. F. Xu Q. Chen M. Peng W. (2013). Glutamate-dependent neuroglial calcium signaling differs between young and adult brain. Science 339, 197–200 10.1126/science.1226740 23307741 PMC3569008

[B148] Takagaki G. (1974). Developmental changes in glycolysis in rat cerebral cortex. J. Neurochem. 23, 479–487 10.1111/j.1471-4159.1974.tb06049.x 4278799

[B149] Torres F. V. Hansen F. Doridio Locks-Coelho L. Souza D. O. (2013). Increase of extracellular glutamate concentration increases its oxidation and diminishes glucose oxidation in isolated mouse hippocampus: reversible by TFB-TBOA. J. Neurosci. Res. [Epub ahead of print]. 10.1002/jnr.23187 23359514

[B150] van den Berg C. J. Garfinkel D. (1971). A stimulation study of brain compartments. Metabolism of glutamate and related substances in mouse brain. Biochem. J. 123, 211–218 5164952 10.1042/bj1230211 PMC1176925

[B151] Vogel R. Hamprecht B. Wiesinger H. (1998). Malic enzyme isoforms in astrocytes: comparative study on activities in rat brain tissue and astroglia-rich primary cultures. Neurosci. Lett. 247, 123–126 10.1016/S0304-3940(98)00290-0 9655608

[B152] Watanabe H. Passonneau J. V. (1973). Factors affecting the turnover of cerebral glycogen and limit dextrin in vivo. J. Neurochem. 20, 1543–1554 10.1111/j.1471-4159.1973.tb00272.x 4198154

[B153] Watkins J. C. (2000). L-Glutamate as a central neurotransmitter: looking back. Biochem. Soc. Trans. 28, 297–309 10.1042/0300-5127:0280297 10961913

[B154] Westergaard N. Drejer J. Schousboe A. Sonnewald U. (1996). Evaluation of the importance of transamination versus deamination in astrocytic metabolism of [U-^13^C]glutamate. Glia 17, 160–168 10.1002/(SICI)1098-1136(199606)17:2<160::AID-GLIA7>3.3.CO;2-S 8776582

[B155] Wilbur D. O. Patel M. S. (1974). Development of mitochondrial pyruvate metabolism in rat brain. J. Neurochem. 22, 709–715 10.1111/j.1471-4159.1974.tb04284.x 4407094

[B156] Wilson D. A. Sullivan R. M. (1991). Olfactory associative conditioning in infant rats with brain stimulation as Reward. II. Norepinephrine mediates a specific component of the bulb response to reward. Behav. Neurosci. 105, 843–849 10.1037/0735-7044.105.6.843 1663758 PMC1885986

[B157] Wysmyk-Cybula U. Faff-Michalak L. Albrecht J. (1991). Effects of acute hepatic encephalopathy and in vitro treatment with ammonia on glutamate oxidation in bulk-isolated astrocytes and mitochondria of the rat brain. Acta Neurobiol. Exp. (Wars.) 51, 165–169 1687972

[B158] Xu J. Song D. Xue Z. Gu L. Hertz L. Peng L. (2013). Requirement of glycogenolysis for uptake of increased extracellular K^+^ in astrocytes: potential implications for K^+^ homeostasis and glycogen usage in brain. Neurochem. Res. 38, 472–485 10.1007/s11064-012-0917-8 23232850

[B159] Yu A. C. H. (1984). Interactions Between Neurons and Astrocytes in Glutamate and Glutamine Metabolism. Ph.D. thesis, University of Saskatchewan, Saskatoon, SK

[B160] Yu A. C. Drejer J. Hertz L. Schousboe A. (1983). Pyruvate carboxylase activity in primary cultures of astrocytes and neurons. J. Neurochem. 41, 1484–1487 10.1111/j.1471-4159.1983.tb00849.x 6619879

[B161] Yu A. C. Schousboe A. Hertz L. (1982). Metabolic fate of ^14^C-labeled glutamate in astrocytes in primary cultures. J. Neurochem. 39, 954–960 10.1111/j.1471-4159.1982.tb12598.x 6126524

[B162] Zwingmann C. Brand A. Richter-Landsberg C. Leibfritz D. (1998). Multinuclear NMR spectroscopy studies on NH_4_Cl-induced metabolic alterations and detoxification processes in primary astrocytes and glioma cells. Dev. Neurosci. 20, 417–426 10.1159/000017339 9778580

